# Influence of source directivity and site effects of 2003 Tokachi-oki earthquake on the generation of high PGA in the near-fault zones

**DOI:** 10.1038/s41598-022-16085-7

**Published:** 2022-07-15

**Authors:** Olga V. Pavlenko

**Affiliations:** Schmidt Institute of Physics of the Earth, Russian Academy of Sciences, B. Gruzinskaya 10, Moscow, 123242 Russia

**Keywords:** Natural hazards, Solid Earth sciences

## Abstract

We study source directivity and site effects of 2003 Tokachi-oki earthquake (Japan, *M*_*w*_ ~ 8.3) and their influence on the distribution of peak ground accelerations (PGA) in the near-fault zones are studied. Based on records of KiK-net vertical arrays, models of soil behavior are constructed, i.e. vertical distributions of stresses and strains induced in soil layers by strong motion. We use the method of Pavlenko and Irikura (2003), previously applied for studying soil behavior during 1995 Kobe, 2000 Tottori, and 2011 Tohoku earthquakes. During the Tokachi-oki earthquake, we did not find a widespread nonlinearity of soft soil behavior. Manifestations of soil nonlinearity were observed at sites closest to the source; at remote sites where high PGA were recorded, soil behavior was virtually linear, and shear moduli in soils increased till the moments of the highest intensity of motion, then decreased. The shapes of acceleration time histories at remote sites indicate directivity effects: seismic waves radiated by the crack tip during its propagation along a section of the fault plane came to the stations simultaneously. Soil hardening occurred at these sites that increased amplification and PGA on the surface. Similar effects were observed during 2011 Tohoku earthquake; evidently, they can occur during future strong earthquakes.

## Introduction

In recent decades, the requirements for the accuracy and reliability of seismic hazard assessment have increased due to the construction of nuclear power plants, high-rise buildings and other complicated engineering structures over the world, including areas of high seismic activity. Networks for seismic observations are developed, allowing for quick accumulation of strong motion records. Dense seismic networks K-NET (~ 1000 surface accelerometers) and KiK-net (~ 800 vertical arrays) were deployed in Japan at the end of the 1990s, and now we can study in detail the effects of strong earthquakes in the near-fault zones. The observations revealed rather complicated distributions of PGA in the near-fault zones of large earthquakes with extended sources. Thus, during the 2011 Tohoku earthquake PGA exceeding 1 g were recorded near the source, as well as at remote sites far from the epicenter.

Evidently, the distributions of PGA in the near-fault zones of large earthquakes should be dependent on the source effects. Since the 1980s, seismologists study the effects of directivity of seismic radiation of extended seismic sources. Archuletta and Hartzell^1^ noted that the database of strong motion records from sites near moderately large earthquakes increases, and “to analyze such records, a clear understanding is needed of the complications that arise when one is no longer in the far-field but situated close to a finite rupture”. When the receiver is near the source, so that the radiation originates over some area, the receiver distance, radiation pattern, and arrival times of P- and S- waves are ill defined, and “the ground motion cannot be interpreted using the same approach that was appropriate for the far-field”. By means of numerical simulation of high-frequency ground motion, they revealed a strong influence of directivity effects on the acceleration in the near-source zones of the 1979 Imperial Valley earthquake, where PGA ~ 1195 cm/s^2^ was recorded.

Sommerville et al.^2^ concluded that directivity effects occur when the propagation of rupture toward a site at a velocity that is almost as large as the shear wave velocity causes most of the seismic energy from the rupture to arrive in a single large pulse of motion, which represents the cumulative effect of almost all of the seismic radiation from the fault; this should be taken into account when predicting the ground motion in the near-fault zones. Abrahamson^3^ suggested to explicitly include directivity effects in the attenuation relations for either probabilistic or deterministic analyses in cases when long period structures such as bridges are located near faults with high activity rates. Si and Midorikawa^4^ found the influence of the rupture directivity on attenuation relationships. Kalkan and Kunnath^5^ investigated the consequences of characteristics of near-fault ground motions on the seismic response of steel moment frames; they attempt to collate analytical evidence from nonlinear dynamic analyses on possible structural effects of strong velocity pulses contained in near-fault ground motions.

Rupture propagation in the fault planes of strong earthquakes can be accompanied by the generation of shock wave fronts (as a limiting case of directivity effects), when a rupture accelerates to high speeds at extended homogeneous and fairly smooth sections of the fault plane. Such phenomena were observed during the 1999 Turkey earthquakes^6^, 2001 Kunlunshan earthquake^7,8^, 2001 Kokoxili earthquake^9^, 2002 Denali (Alaska) earthquake^10^, and some others. Also, it was concluded that large subduction-type earthquakes, such as, the 2004 Sumatra earthquake can have a rupture front, which can reach speeds as high as those during the Kunlunshan earthquake^8^.

In my previous paper^11^, abnormally high accelerations exceeding 1 g are analyzed recorded during the 2011 Tohoku earthquake at KiK-net sites TCGH16, IBRH11, FKSH10 and others located far enough from the epicenter. These high PGA can be explained by the following mechanism: the crack in the fault plane of the Tohoku earthquake propagated at a speed of ~ 4 km/s towards these sites along a rather long (~ 120 km) section of the fault plane, so that seismic waves radiated by the crack tip came to the sites simultaneously. Shock wave fronts were generated that produced some additional compression of soils at these sites and increased amplification of seismic waves in soil layers and high PGA on the surface as a result.

**Figure 1:**
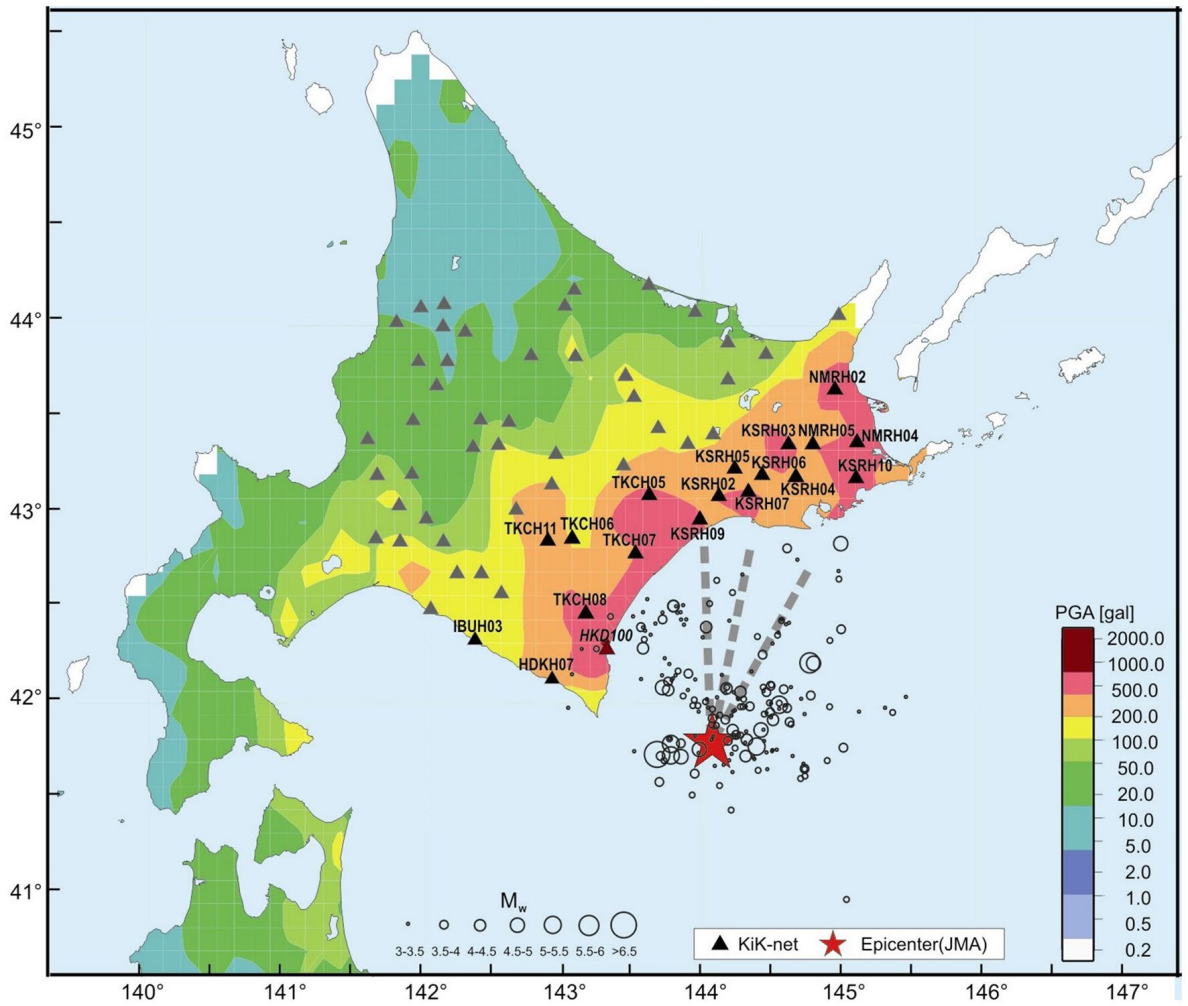
Map showing the distribution of PGA recorded during the 2003 Tokachi-oki earthquake (NIED data^21^), locations of the main shock (star), aftershocks (circles) (*M*_*w*_ > 3) recorded in 24 h after the main shock (JMA data), and KiK-net stations (triangles). The dotted lines show horizontal projections of the rupture propagation towards KSRH09, KSRH06, and KSRH10 sites.

The distribution of PGA in the near-fault zones of the 2003 Tokachi-oki earthquake shows similar mosaic pattern as observed during the Tohoku earthquake (Fig. 1). The highest accelerations were recorded close to the epicenter, at HKD100 (988.4 Gal), HKD086 (800.6 Gal), and HKD092 (672.7 Gal) sites, as well as at remote sites KSRH03 (884.4 Gal), KSRH10 (613.0 Gal), NMRH02 (607.2 Gal), HKD066 (591.1 Gal), and HKD075 (560.8 Gal). The researchers, who studied the Tokachi-oki earthquake, Yagi^12^ and Koketsu et al.^13^, attribute these high accelerations to the directivity effects.

The subduction-type 2003 Tokachi-oki earthquake occurred off the coast of Hokkaido, Japan, on September 26, with the moment magnitude *M*_*w*_ ~ 8.0–8.3 and the focal depth ~ 18–42 km. According to the various authors, the maximum slip on the fault plane varied from ~ 4.3 to ~ 13 m^14^. The earthquake caused extensive damage, landslides, soil liquefaction and a tsunami up to 4 m in height.

The source process was studied by many research groups. Yamanaka and Kikuchi^15^ estimated source parameters based on teleseismic P- and SH- waves and concluded that the rupture propagated northward from a shallow to a deep region. Their results suggest that the 2003 Tokachi-oki earthquake was a recurrent event of the 1952 Tokachi-oki earthquake (*M*_*s*_ ~ 8.2).

In papers by Yagi^12^, Koketsu et al.^13^, and Honda et al.^16^, source parameters were estimated based on teleseismic body waves and strong motion records^12^, on strong motion records^16^, on geodetic data and strong motion records^13^. The authors used strong motion records at K-NET and KiK-net sites located in the near-fault zones, ~ 12–15 sites. Yagi^12^ found that a rupture front velocity of 4.5 km/s in the middle and deep parts of the fault shows the best fit to the observations. Honda et al.^16^ identified three main slip areas, such as, around the hypocenter, in the northwest part of the fault (with a maximum slip of 5.9 m), and in the northeast part of the fault, the longest in time. Koketsu et al.^13^ estimated the maximum slip on the fault plane as 7.1 m, and the rupture propagation velocity in the upper part of the fault as supershear—4.3 km/s, decreasing to S-wave velocity in the middle and deep parts of the fault. They obtained simple slip patterns and near-supershear rupture that may imply the maturity of the Hokkaido subduction zone around the source region.

Miura et al.^17^ estimated coseismic and postseismic slips based on GPS data and concluded that the major postseismic slip extended from south of the epicenter about 180 km to the northeast. Rubinstein et al.^18^ based on repeating earthquake sequences identified velocity changes caused by the earthquake. They found significant velocity reductions up to 0.3% close to the receivers, evidently caused by damage to near-surface materials by nonlinear strong motion. The second region where seismic velocities were reduced as a result of the main shock was the rupture zone. Hatayama^19^ studied long-period (4–8 s) waves induced by strong motion in the Yufutsu sedimentary basin (IBUH03 station area, Fig. 1) that caused destructions of oil storage tanks.

Nozu and Irikura^20^ inverted with empirical Green’s functions the waveforms of the 2003 Tokachi-oki earthquake at 39 KiK-net sites and studied in detail high-frequency components (up to ~ 1 Hz). They identified the locations of three strong motion generation areas (SMGA) on the fault plane, that well agree with asperities identified from the inverted results using low-frequency (< 0.2 Hz) ground motions plus geodetic data and tsunamis. They also redefined the location and depth of the hypocenter.

Robinson and Cheung^14^ reviewed source parameters obtained by many researchers and estimated the parameters themselves based on broadband SH-wave seismograms; they concluded that the rupture occurred at a depth of ~ 18 km (in agreement with Yagi^12^), and the maximum slip was about 12 m.

As we can see from the review, studies of the 2003 Tokachi-oki earthquake performed by various authors mainly concerned the source mechanism; the researchers did not study the distribution of peak ground accelerations and velocities in the near-fault zones, though their distribution was rather complicated and high accelerations were recorded. That is, the interesting and important site effects in the near-fault zones remained unexplored.

The researchers did not comment the occurrence of high accelerations at remote stations KSRH03, KSRH10, NMRH02 and others. The only work by Nozu and Irikura^20^ contains some discussion about the records at KSRH03 site. The authors note that their simulations give lower amplitudes at KSRH03 site than those observed, and to correct this, introduce SMGA at the edge of the fault plane just near KSRH03, which slightly corrects the situation.The purpose of this work is to explain the distribution of PGA in the near-fault zones of the 2003 Tokachi-oki earthquake. To do this, we study soil behavior at KiK-net sites in the near-fault zones and changes in shear moduli in soil layers during strong motion. Then we interpret the obtained models of soil behavior taking into account the effects of source directivity. Many KiK-net sites in the near-fault zones are located on soft soils, and their records provide valuable information about soft soil behavior during a strong earthquake of magnitude *M*_*w*_ ~ 8.3, possessing long-lasting motion.

In this paper we mean the ‘near-fault zone’ as the area near the fault, where accelerations of ~ 200 Gal and more were recorded (Fig. 1). The dimensions of this area are close to the dimensions of the earthquake source, which estimates vary from 90 km × 70 km^12^ to 200 km × 160 km^29^, according to different authors.

## Methods and data

Figure 1 shows the location of the main shock of the Tokachi-oki earthquake, the aftershocks occurred in the first 24 h after the mainshock, and the locations of 18 KiK-net stations where soil behavior was studied. Table 1 provides information on the studied sites: the coordinates, epicentral distances, soil conditions (*V*_*s30*_, the thickness of surface softer layers *d* and S-wave velocities in the layers *V*_*s*_), recorded PGA. As seen from the Table, KiK-net stations recorded the highest PGA were located on soft soils.

**Table 1:** Information on the KiK-net sites, for which models of soil behavior during the 2003 Tokachi-oki earthquake were constructed.

Site code	Latitude (°)	Longi-tude (°)	Subsur-face soils: *d*/*V*_*s*_	*V*_*s30*_, m/s	Epicen-tral distance, km	PGA on surface, Gal	Depth of borehole, m
TKCH08	42.49	143.15	4/130	353	80	539.8	103
HDKH07	42.13	142.92	2/190	495	84	207.7	104
TKCH07	42.81	143.52	38/120	140	89	414.0	103
KSRH09	42.99	143.98	10/118	230	102	454.6	103
TKCH06	42.89	143.06	2/130	300	115	192.6	230
KSRH02	43.11	144.12	18/162	219	116	462.0	108
TKCH05	43.12	143.62	6/140	337	118	497.4	103
KSRH07	43.14	144.33	14/125	204	122	538.0	225
TKCH11	42.87	142.88	1/150	459	124	316.2	103
KSRH06	43.22	144.43	2/90	326	133	394.5	240
KSRH05	43.26	144.23	4/100	389	133	347.6	333
KSRH04	43.21	144.68	30/189	189	140	362.2	243
KSRH03	43.38	144.63	16/192	250	155	884.4	110
KSRH10	43.21	145.12	36/224	213	159	613.0	258
NMRH05	43.39	144.80	20/174	209	161	406.6	223
NMRH04	43.40	145.12	146/258	168	175	518.3	219
IBUH03	42.65	141.86	76/174	111	182	377.9	156
NMRH02	43.68	144.96	10/181	315	195	607.2	106

Simultaneous records of two accelerometers at KiK-net sites allow us to simulate the behavior of soil layers during strong motion from the surface down to the location of the deep device. Models of soil behavior, such as, vertical distributions of stresses and strains in soil layers changing in time during strong motion are constructed using the method by Pavlenko and Irikura^22^. The method is based on processing records of seismic vertical arrays, and it was previously applied to study soil behavior during the 1995 Kobe earthquake^22,23^, 2000 Tottori earthquake^24^, and 2011 Tohoku earthquake^25^. The constructed models of soil behavior illustrate the behavior of different soil layers during strong ground motion.

We calculate the propagation of vertically incident shear waves in the overlying system of horizontal soil layers; the algorithm of nonlinear analysis by Joyner and Chen^26^ is used. To describe the behavior of soil layers, we apply nonlinear stress–strain relations of ‘soft’ type (declining to the strain axis at large strains) or ‘hard’ type (declining to the stress axis at large strains); the type is selected based on the composition of the layers and their saturation with water and accounting for the available information, like liquefaction in the upper layers or spiky waveforms. Series of soft- or hard-type stress–strain relations of various shapes are generated and tested to find the relations showing the best fit to the observations on the surface.

Records of the deep device of the vertical array serve as input to soil layers; to account for possible changes in soil behavior during strong motion, the input is divided into small time intervals. Calculations are performed successively, interval by interval. Within each interval, the stress–strain relations are assumed to be stationary, and vary for different intervals.

At all the studied KiK-net sites soil behavior was described by hard-type stress–strain relations down to the location of the deep device; the best-fit nonlinear stress–strain relations were selected from series of 250 parametric curves. Records of two horizontal components were analyzed, and calculations were performed in successive time intervals of 1.5 s duration. Time interval of 1.5 s was chosen empirically as the optimal one: on one hand, it is long enough to reliably choose the best-fit stress–strain relation (for shorter time intervals several relations are usually suitable), on the other hand, it is quite short so that we can consider soil behavior stable enough to describe it by one stress–strain relation.

The profiling data required for calculations, such as, the composition and thickness of soil layers and P- and S-wave velocities were taken from the website^27^; the density, shear stress in failure *τ*_*max*_, and attenuation were selected based on the composition and depth of the layers. In the calculations, stress–strain relations normalized in the manner suggested by Hardin and Drnevich^28^ were used: stresses and strains were multiplied by 1/*τ*_*max*_ and *G*_*max*_/*τ*_*max*_, respectively, where *G*_*max*_ is the low-strain modulus. The differences in the behavior of soil layers result from the differences in *τ*_*max*_ and *G*_*max*_ values in the layers.

The best-fit relations were selected based on the deviations of the simulated acceleration time histories from the recorded ones. The deviations were calculated as sums of the mean square point-by-point deviations, accounting for the agreement in peak accelerations and frequency contents of the simulated and recorded accelerogramms. The method is described in more detail by Pavlenko and Irikura^22^.

Thus, soil behavior at KiK-net sites during the earthquake was simulated in 50 1.5-s time intervals (75 s of strong motion), i.e., stress–strain relations in soil layers were found that reflect the features of soil behavior during the earthquake.

The constructed models allow us to estimate changes in shear moduli in soil layers induced by strong motion. The models of soil behavior represent groups of hysteretic stress–strain curves describing the cycles of loading and unloading of soil layers, and shear moduli were estimated as the ratios of the normalized average stresses to the normalized average strains calculated over all the curves within each 1.5-s interval and then averaged over the entire soil thickness, from the surface down to the location of the deep device.

To trace the effects of directivity, acceleration time histories were analyzed (records of deep devices, to eliminate soil response) in different directions at different epicentral distances.

## Results

**Figure 2:**
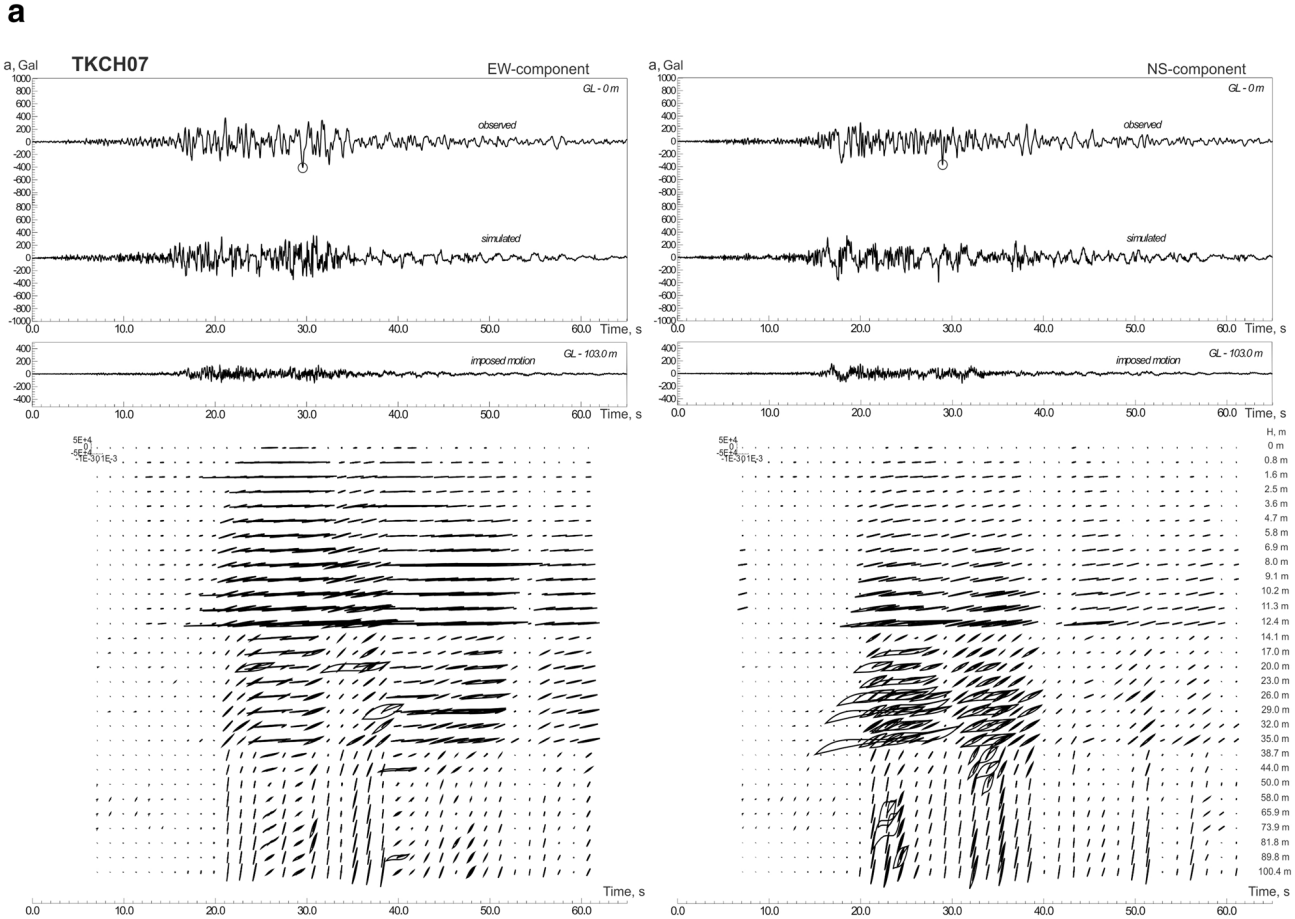

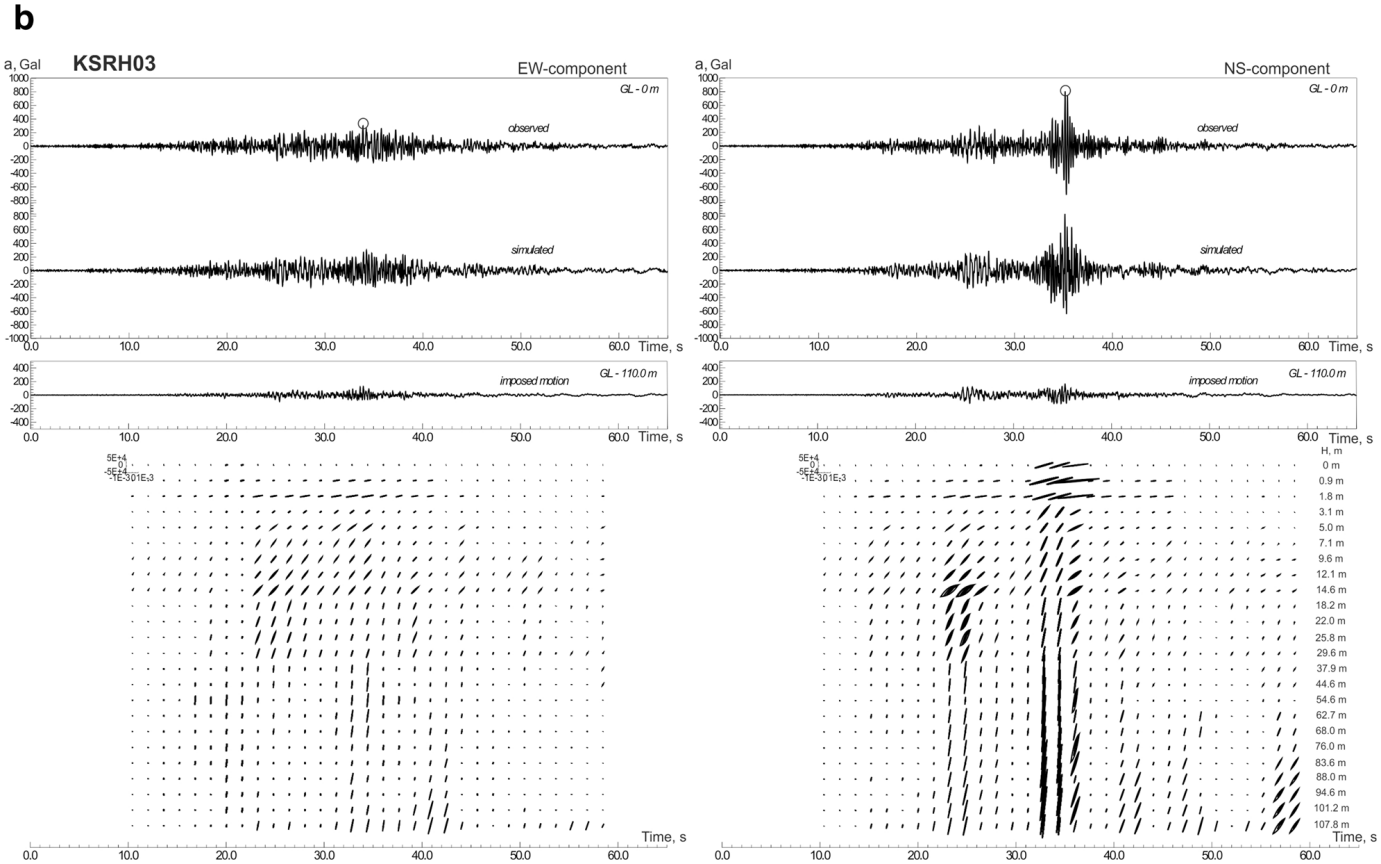

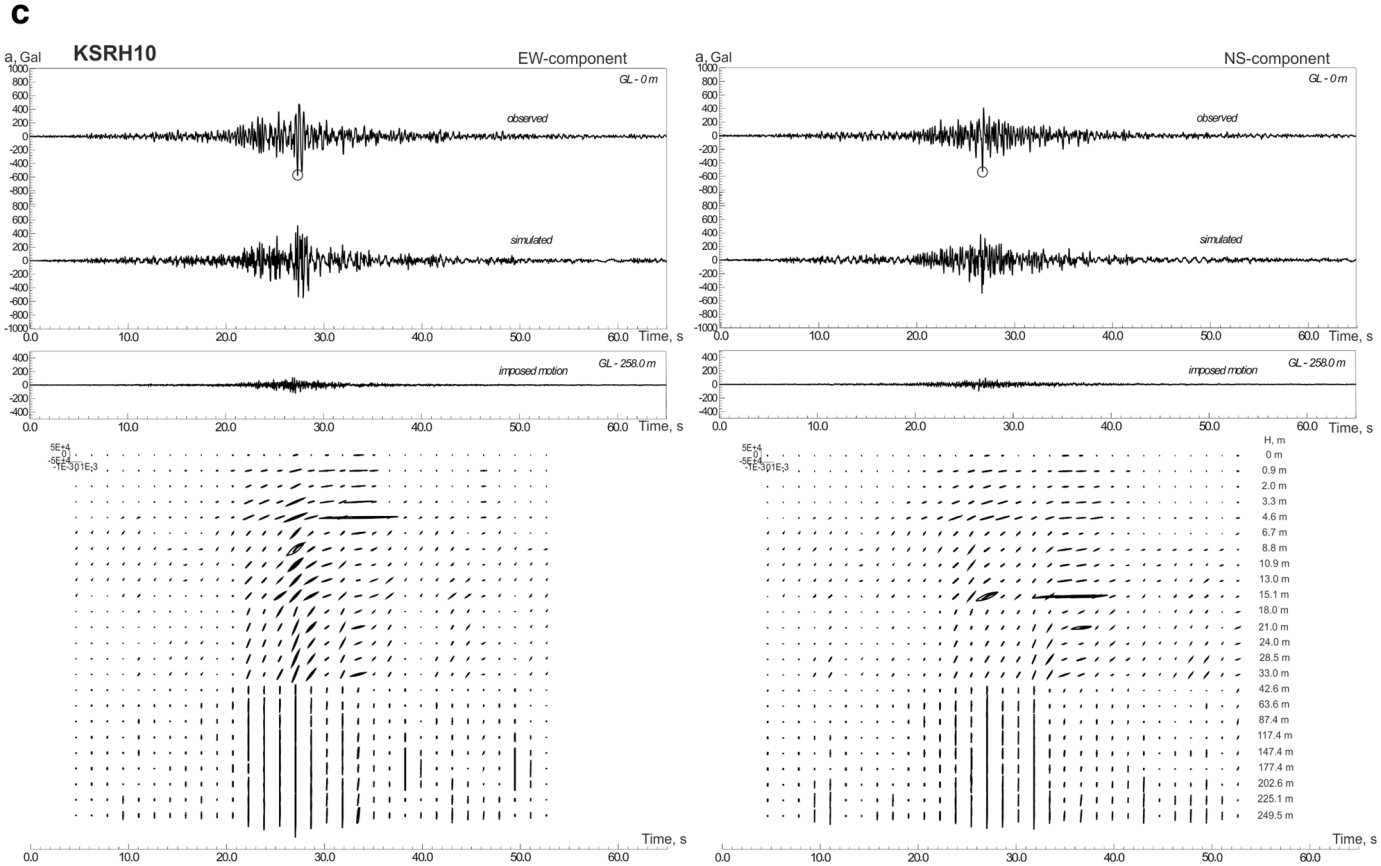
Acceleration time histories of the 2003 Tokachi-oki earthquake, observed and simulated, and estimated stress–strain relations in soil layers, changing with time during strong motion: (**a**) at TKCH07 site; (**b**) at KSRH03 site; (**c**) at KSRH10 site. Stresses are given in Pa, strains in strain. Peak ground accelerations are marked by small circles.

The constructed models of soil behavior at KiK-net softer and denser soil sites are shown in Figs. 2a–c, S1a–h and S2a–f. We consider soil sites with *V*_*s30*_ > 300 m/s (Table 1) as denser soil sites; these are mostly gravelly soils in the upper layers. Softer soil sites (*V*_*s30*_ < 300 m/s) contain silts, sands, clays, and volcanic ash in the upper layers.

As seen from the figures, a fairly good agreement is obtained between the calculations and observations (Figs. 2, S1, S2). Differences in the behavior of softer and denser soils are observed only at sites located close to the epicenter: TKCH07, KSRH09 and TKCH06 sites indicate nonlinear behavior of soft soils (Figs. 2a, S1a,b), whereas, TKCH08, HDKH07, TKCH05, TKCH11 sites demonstrate virtually linear behavior of denser soils (Fig. S2a–d).

The behavior of soft soils becomes more linear with increasing distance from the fault plane (Fig. 2b,c, S1d–h). At TKCH07 and KSRH09 sites we see signs of liquefaction of sandy water-saturated soils in the upper 14 m (at TKCH07) and in the upper 6 m (at KSRH09) and nonlinear behavior (demonstrated by hysteretic curves) of sandy soils in deeper layers in time intervals 20–40 s from the beginning of strong motion (Figs. 2a, S1a). Quasi-linear behavior of soft soils at remote sites means low nonlinear damping, which agrees with high accelerations recorded at some of them. At KSRH07, KSRH03, KSRH10, and NMRH02 sites at the moments of the highest intensity of strong motion, soil behavior is described by quasi-linear stress–strain relations indicating soil hardening (Fig. 2b,c, S1d,h). Similar behavior of soft soils was observed during the 2011 Tohoku earthquake at remote sites located ~ 280–310 km from the epicenter^25^.

**Figure 3:**
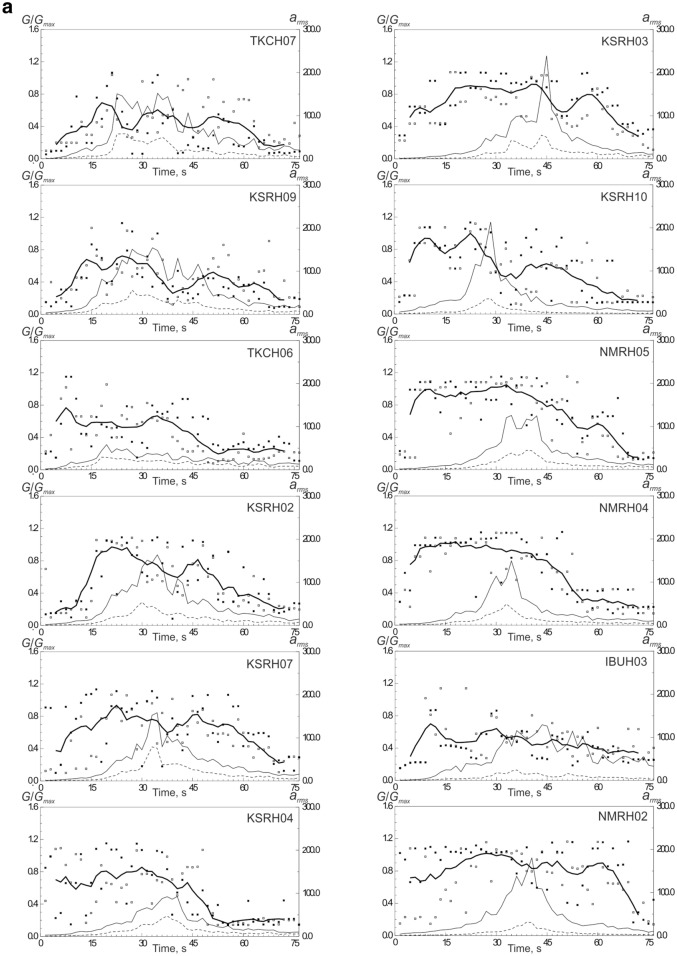

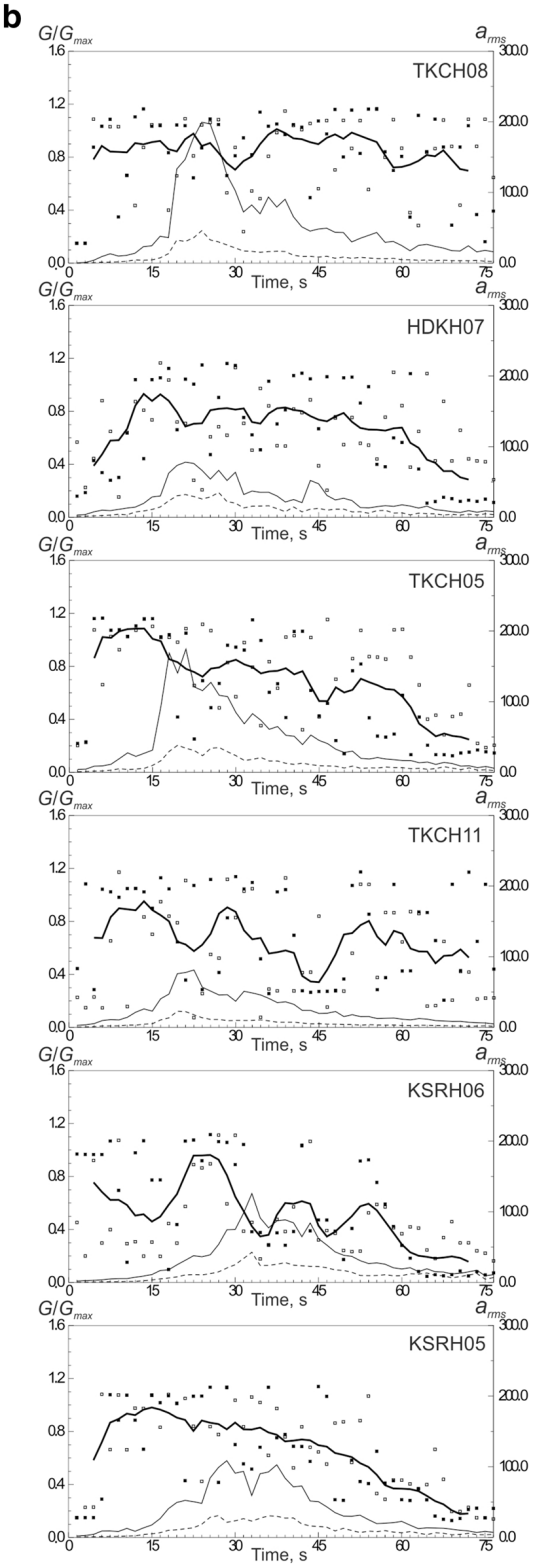
Changes of shear moduli in soil layers at the studied KiK-net sites during the Tokachi-oki earthquake (75 s of strong motion): (**a**) at sites with softer subsurface soils, (**b**) at sites with denser subsurface soils. Points indicate estimated shear moduli in successive time intervals at two horizontal components (white and black points mark EW and NS components), and thick lines show these values smoothed and averaged over two horizontal components. Thin lines are the intensities of motion on the surface (solid lines) and at depths of locations of deep devices (dash lines).

The estimates of shear moduli in soil layers changing in time during strong motion (75 s) at softer and denser soil sites are shown in Fig. 3a,b. The estimates should be considered as approximate ones, because they are based on the models of soil behavior, approximating the actual soil behavior; however, we can notice some regularities. As seen from the figures, the shear moduli grow and fall during strong motion, i.e., soils (both, soft and dense) experience hardening and softening; each cycle lasts ~ 10–15 s. At the majority of sites we observe soil softening after reaching the highest intensity of strong motion (as clearly seen at sites KSRH09, KSRH02, KSRH07, KSRH03, KSRH10, TKCH08, and others—Fig. 3a, b).

At the initial part of strong motion it is difficult to estimate shear moduli correctly, because the motions are still weak (several stress–strain relations are suitable); we can obtain fairly reliable estimates after ~ 15–20 s of strong motion, and at these moments shear moduli are usually high (~ 0.8), and they reduce down to ~ 0.2 in the final parts of motion.

At remote soft soil sites recorded high PGAs, such as, KSRH04, KSRH10, NMRH05, NMRH04 (Fig. 1, Table 1), shear moduli variations are rather simple: they are high (~ 0.8–1.0) at the beginning of strong motion and reduce to ~ 0.2 after reaching the highest intensity of motion (Fig. 3a). Similar behavior was observed at remote sites recorded high accelerations during the 2011 Tohoku earthquake^25^.

During the Tohoku earthquake, such stations with soft soils showed a stepwise decrease of the predominant frequencies of motion on the surface from ~ 4 to ~ 2 Hz after reaching the highest intensity of motion^25^. In the near-fault zones of the Tokachi-oki earthquake many KiK-net sites were located on soft soils, and it was interesting to check, if a similar decrease of frequencies occurred at these sites. Time–frequency diagrams of motion at soft soil sites KSRH04, KSRH03, KSRH10, and NMRH04 (Fig. S3) show some indications of decreasing the predominant frequencies after reaching the highest intensity of motion from 6–8 to ~ 1–2 Hz.

Thus, during the 2003 Tokachi-oki earthquake, we observe phenomena similar to those observed during the 2011 Tohoku earthquake, such as, nonlinear behavior of soft soils at sites located close to the epicenter; at remote sites recorded high PGA—virtually linear behavior of soft soils, their hardening at the moments of the highest intensity of motion and softening with decreasing the intensity of motion. Soft soil hardening at remote sites (as in the case of the Tohoku earthquake^11^) indicates the presence of some additional compression acting on the soils, and compressed soils behave more linearly under strong motion. The compression may be caused by a shock wave generated in the source of the Tokachi-oki earthquake.

As shown in my previous paper^11^, these features of soil behavior at remote sites may result from the directivity effects occurred during the crack propagation in the extended source of the Tohoku earthquake. The directivity effects (their limiting case, a shock wave) were traced by changes of the waveforms of the acceleration time histories with epicentral distance. Peak accelerations have not noticeably changed with distance, while the duration of strong motion decreased. At epicentral distances of ~ 270–310 km, the strong motion duration reached its minimum, while PGA increased (at FKSH10, TCGH10, TCGH16, and IBRH11 sites); at larger distances PGA and intensities of motion sharply fell.

Similarly, during the 2003 Tokachi-oki earthquake directivity effects were essential, as noted, for example, by Koketsu et al.^13^ and Honda et al.^16^. High PGA were recorded in coastal areas to the northwest of the epicenter, at stations HKD100 (988.4 Gal), HKD086 (800.6 Gal), and HKD092 (672.7 Gal). Honda et al.^16^ concluded that directivity effects of the largest asperity might have increased the amplitudes of seismic waves incident to the Yufutsu sedimentary basin, where oil storage tanks were severely damaged. Koketsu et al.^13^ performed joint inversion of strong motion and geodetic data and found that the rupture propagated at a supershear speed on the upper part of the fault plane near the hypocenter, and this supershear (*V*_*r*_ > *V*_*s*_) or near-supershear (*V*_*r*_ ∼ *V*_*s*_) situation resulted in directivity effects on P-waves and S-waves. The effects are confirmed by long-period pulses in observed velocigrams at sites located to the north of the epicenter (TKCH08, TKCH10, TKCH11, and ABSH04).

At the same time high PGA ~ 1* g* were recorded at remote sites KSRH03 (884.4 Gal), KSRH10 (613.0 Gal), NMRH02 (607.2 Gal), HKD066 (591.1 Gal), and HKD075 (560.8 Gal) located northeast of the epicenter, where these high accelerations can also be associated with directivity effects.

**Figure 4:**
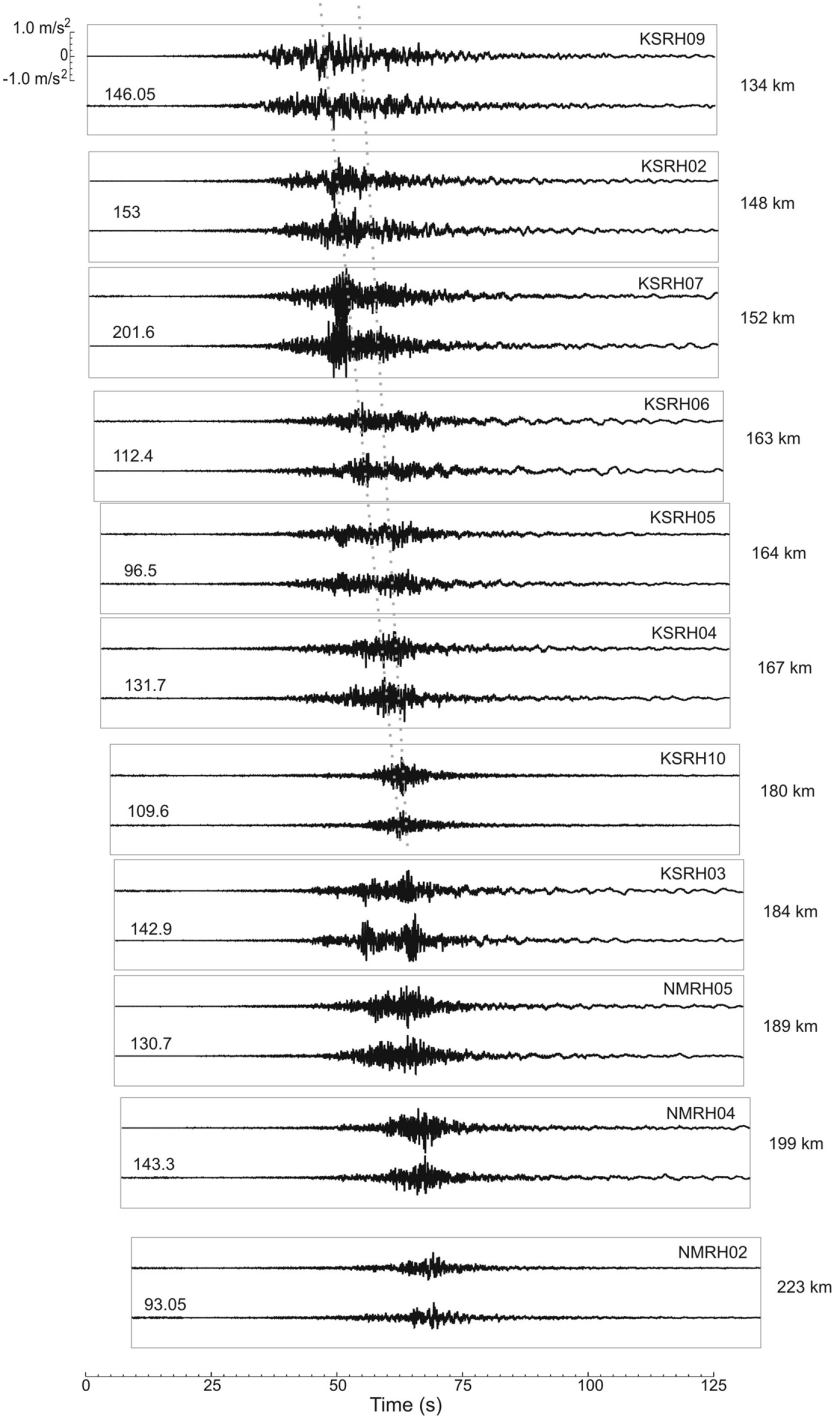
Acceleration time histories (EW and NS components, depth records) of the Tokachi-oki earthquake at KiK-net sites located to the north of the epicenter. For each site, the epicentral distances (on the right) and PGA in cm/s2 (on the left) are shown. Dotted lines show the decrease of the duration of strong motion with distance.

Figure 4 shows horizontal components of the acceleration time histories of the Tokachi-oki earthquake (records of deep devices) at KiK-net sites located north and northeast of the epicenter at various epicentral distances. Similar to Tohoku earthquake, we observe a decrease of the duration of strong motion with distance, while PGA have not changed significantly. The minimal duration of strong motion is observed at sites KSRH10, KSRH03 (the 2-d group of intense waves), and NMRH02, where the highest PGA were recorded (Fig. 4, Table 1). At KSRH03 and at neighboring NMRH05 site intense motion is seen before the analyzed peaks, which may be related to some heterogeneities in this area.

**Figure 5:**
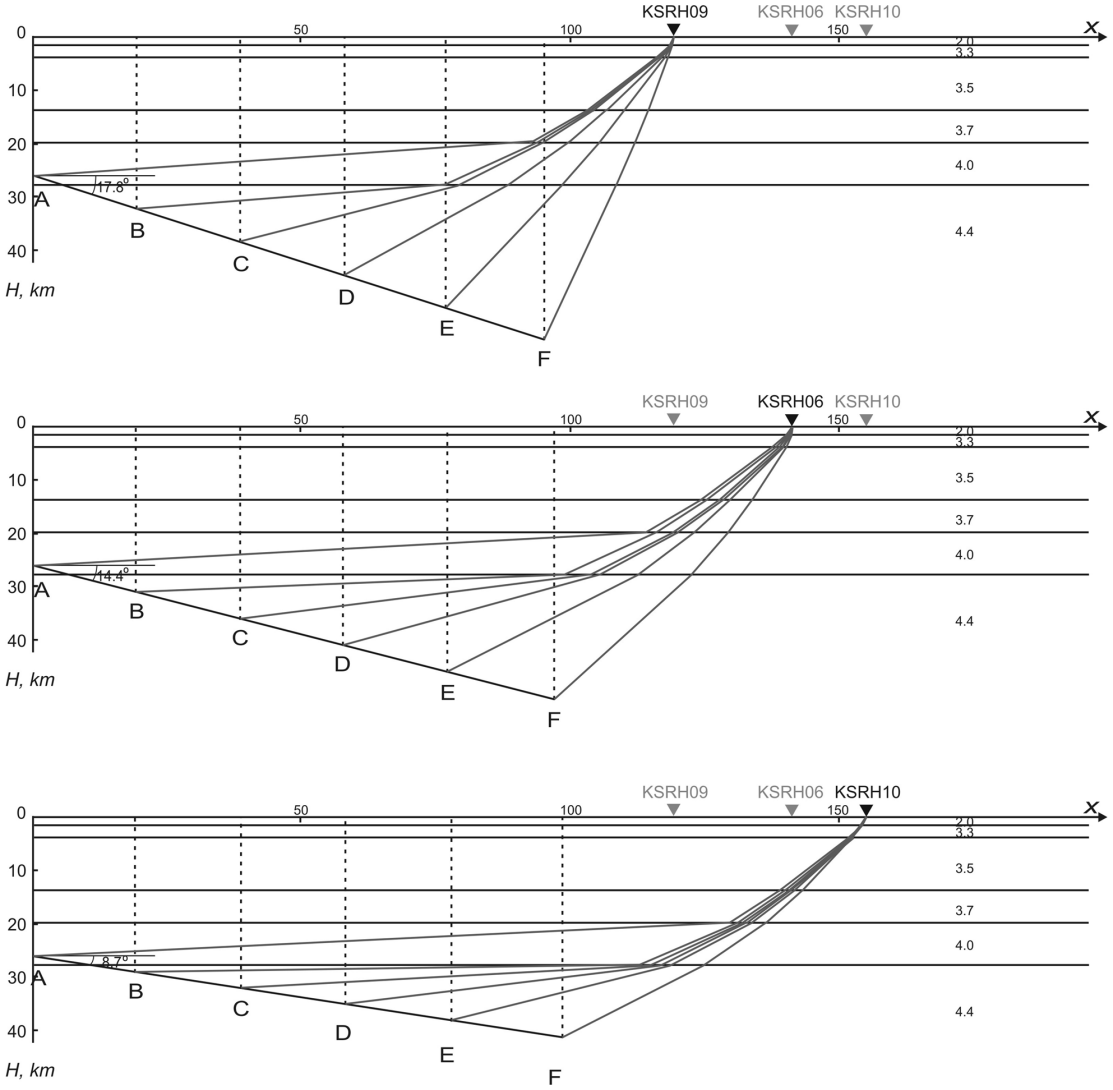
The cross-section of the fault plane of the Tokachi-oki earthquake, locations of KiK-net sites and seismic wave paths. The numbers indicate S-wave velocities in the layers in km/s; velocity structure model is taken from^13^. S-wave propagation: (**a**) to KSRH09, (**b**) to KSRH06, (**c**) to KSRH10.

To check the influence of the directivity effects on the records of stations located to the northeast of the epicenter, simple calculations were performed (Tables 2, 3, 4). The propagation of seismic waves radiated by the crack moving from the hypocenter towards three stations, KSRH09, KSRH06, and KSRH10 is calculated. Figure 5 schematically shows the cross-section of the fault plane of the 2003 Tokachi-oki earthquake and the locations of the stations, and Tables 2, 3 and 4 present calculations showing the difference between seismic wave propagation to the three stations. Superposition of seismic waves could occur at KSRH10 site, i.e., seismic waves radiated by the crack tip during a certain period of time came to the site almost simultaneously, whereas they came to KSRH09 site located closer to the fault plane sequentially, no superposition occurred. KSRH06 is an intermediate case.

**Table 2:** Duration of strong motion that was observed at station KSRH09 as a result of crack propagation over a certain part of the fault plane during the Tokachi-oki event (*l* ~ 100 km, *α* ~ 17.8°, *V* ~ 4.3–4.0 km/s).

The time moment of S-wave radiation by a crack tip (s)	The projection of the distance between the crack tip and KiK-net station on the X axis (km)	The depth of the crack tip *h* (km)	The S-wave travel time from the crack tip to the KiK-net station (s)	The time of S-wave arrival to the KiK-net station (s)
0 (A)	119.5	26	33.12	33.12
4.65 (B)	100.46	32.11	27.50	32.15
9.3 (C)	81.41	38.23	23.40	32.7
13.95 (D)	62.37	44.34	19.82	33.77
18.95 (E)	43.33	50.46	17.13	36.08
23.95 (F)	24.29	56.57	15.72	39.67

The duration of strong motion: 7.52 s.

**Table 3:** Duration of strong motion that was observed at station KSRH06 as a result of crack propagation over a certain part of the fault plane during the Tokachi-oki event (*l* ~ 100 km, *α*  ~ 14.4°, *V* ~ 4.3–4.0 km/s).

The time moment of S-wave radiation by a crack tip (s)	The projection of the distance between the crack tip and KiK-net station on the X axis (km)	The depth of the crack tip *h* (km)	The S-wave travel time from the crack tip to the KiK-net station (s)	The time of S-wave arrival to the KiK-net station (s)
0 (A)	141.6	26	38.63	38.63
4.65 (B)	122.23	30.97	32.46	37.11
9.3 (C)	102.86	35.95	28.11	37.41
13.95 (D)	83.49	40.92	24.02	37.97
18.95 (E)	64.11	45.90	20.34	39.29
23.95 (F)	44.74	50.87	17.42	41.37

The duration of strong motion: 4.25 s.

**Table 4:** Duration of strong motion that was observed at station KSRH10 as a result of crack propagation over a certain part of the fault plane during the Tokachi-oki event (*l* ~ 100 km, *α*  ~ 8.7°, *V* ~ 4.3–4.0 km/s).

The time moment of S-wave radiation by a crack tip (s)	The projection of the distance between the crack tip and KiK-net station on the X axis (km)	The depth of the crack tip *h* (km)	The S-wave travel time from the crack tip to the KiK-net station (s)	The time of S-wave arrival to the KiK-net station (s)
0 (A)	155.4	26	42.07	42.07
4.65 (B)	135.63	29.03	35.43	40.08
9.3 (C)	115.86	32.05	30.94	40.24
13.95 (D)	96.09	35.08	26.56	40.51
18.95 (E)	76.32	38.10	22.28	41.23
23.95 (F)	56.55	41.13	18.32	42.27

The duration of strong motion: 2.19 s.

In the calculations, parameters of the fault plane are used taken from the paper by Koketsu et al.^13^, which partially coincide with the estimates by Yamanaka and Kikuchi^15^: the depth of the hypocenter 25 km and the orientation of the fault plane (230°, 20°); the location of the hypocenter was taken according to JMA: 41.78°, 144.078°.

The locations of the three KiK-net sites relative to the fault plane (Fig. 1) does not allow us to study directivity effects in narrow sectors as done for the Tohoku earthquake^11^; the calculations were performed for three directions shown by the dotted lines in Fig. 1, aimed at KSRH09, KSRH06, and KSRH10 sites. The researchers studied the source process of the 2003 Tokachi-oki earthquake point out a simple model of slip distribution over the fault plane and radial propagation of ruptures from the hypocenter. Therefore, we assume a radial rupture front in the sector marked by the dotted lines (Fig. 1).

Travel times to the stations (Tables 2, 3, 4) are calculated based on the distances between the crack tip and the stations and the 6-layer velocity structure model taken from the paper by Koketsu et al.^13^ (Fig. 5). The calculations were performed for time moments corresponding to the positions of the crack tip over 20-km intervals (points A, B, C, D, E, F); the total path 100 km. The analyzed section of the fault is marked by dotted lines in Fig. 1, it starts near the hypocenter (according to Koketsu et al.^13^); there is the area of high concentration of aftershocks (Fig. 1). S-wave arrival times to the KiK-net stations were calculated as the sums of the time moments of S-wave radiation by the crack tip and the travel times to the stations. Estimates of the crack propagation velocity obtained by Koketsu et al.^13^ are used: supershear, 4.3 km/s in the upper part of the fault (A–B–C–D) decreasing to S-wave velocity, 4.0 km/s in the middle and deep parts of the fault (D–E–F).

The estimates of strong motion duration at the stations are: ~ 8 s at KSRH09, ~ 4.5 s at KSRH06 and ~ 2 s at KSRH10 (Tables 2, 3, 4), which agrees with the observations. Thus, the distance between the crack tip and the KSRH10 station decreased faster than S-waves propagate, and seismic waves radiated by the crack tip over a long time period reached KSRH10 site almost simultaneously. The waves overlapped and PGA increased while the duration of motion decreased.

## Discussion and conclusions

The model (Fig. 5) and the calculations (Tables 2, 3, 4) should be considered as approximate ones, illustrating the directivity effects from the finite-fault source that lead to high PGA at remote sites.

According to various authors, the fault plane of the Tokachi-oki earthquake was 160 km × 40 km on average (the estimates vary from 90 km × 70 km^12^ to 200 km × 160 km^29^). The researchers agree that the fault had a fairly simple slip distribution that may imply the maturity of the subduction zone around the source region and a near-supershear rupture propagation in the hypocenter area as estimated by Koketsu et al.^13^. High values of rupture propagation velocity, 4.5 km/s in north direction were obtained by Yagi^12^. Earthquakes have repeatedly occurred in this area in the past; they smoothed out asperities and facilitated sliding on the fault.

The distribution of aftershocks occurred during the first days and months after the main shock was studied. The highest aftershock concentrations were observed to the northwest of the hypocenter (where Koketsu et al.^13^ and Yagi^12^ found near-supershear rupture propagation and directivity effects), as well as to the northeast, to KSRH10 site. High concentration of aftershocks is usually associated with the areas of large slip, high stress drop and high rupture velocity, as noticed by Robinson et al.^8^. The major postseismic slip extended from south of the epicenter about 180 km to the northeast^17^.

Considering these facts it seems quite likely that the rupture spread at a speed of ~ 4.3–4.0 km/s radially to the north-northeast from the hypocenter, which produced directivity effects at remote sites KSRH10, KSRH03, NMRH02 and some others. Seismic waves radiated by the crack tip during its propagation over a rather long segment (~ 100 km) of the fault came to remote sites almost simultaneously, which decreased the duration and increased PGA at the inputs to soil layers. Since seismic waves were radiated by a source moving towards the stations, the frequencies of motion increased due to the Doppler effect. The soil layers at the sites experienced hardening at the moments of the maximum intensity of motion, amplification of seismic waves increased, and high PGA were recorded on the surface.

Similar mechanisms worked during the 2011 Tohoku earthquake^11^. During future large earthquakes directivity effects may also be significant, and we may expect generation of high PGA in the near-fault zones.

Notice the different behavior of soft soils during earthquakes with magnitudes *M*_*w*_ ~ 6.5–7.0 and stronger earthquakes with magnitudes *M*_*w*_ ~ 8.0 and higher. In the first case, the duration of strong motion in the near-fault zones does not exceed ~ 15–20 s, and soft soil behavior is substantially nonlinear near the source (and nonlinearity gradually decreases with distance). Shear moduli in soil layers reduce at the beginning and recover at the end of strong motion^23,24^.

During stronger earthquakes with magnitudes *M*_*w*_ ~ 8.0 and higher, strong motion duration reaches tens to hundreds seconds, so that soil layers experience several stages of softening–hardening, reduction and recovering of the shear moduli (as we see from the constructed models of soil behavior and variations of shear moduli). The physical cause of the cyclic soil hardening and softening is not quite clear; probably it is related to the movement of the underground water. On the whole, the behavior of soft soils can be rather linear in the near-fault zones. During the 2003 Tokachi-oki earthquake, shear moduli at sites with softer and denser soils finally reduced at the end of strong motion and did not recover.

## Supplementary Information

Supplementary Information.

## Data Availability

Records of the 2003 Tokachi-oki earthquake and the profiling data are provided by the National Research Institute for Earth Science and Disaster Prevention (NIED^21^) in Japan and can be obtained from the Kyoshin and Kiban-Kyoshin Networks at www. kyoshin.bosai.go.jp.
