# Supplementary material for: Influence of source directivity and site effects of 2003 Tokachi-oki earthquake on the generation of high PGA in the near-fault zones
Source: Sci Rep. 2022 Jul 15;12:12134. doi: 10.1038/s41598-022-16085-7 (PMC9287307; doi:10.1038/s41598-022-16085-7)
Supplement: Supplementary file 1 — Supplementary Information. [file 41598_2022_16085_MOESM1_ESM.docx]

Supplementary Information file for the article:

**Influence of source directivity and site effects of 2003 Tokachi-oki earthquake on the generation of high PGA in the near-fault zones**

Olga V. Pavlenko

Schmidt Institute of Physics of the Earth, Russian Academy of Sciences, B. Gruzinskaya 10, Moscow 123242, Russia

e-mail: olga@ifz.ru, phone: +7 499 254 9025, mobile: +7 915 333 0260

This file is provided to contain all the necessary Figures that could not be included in the main text due to space constraints.


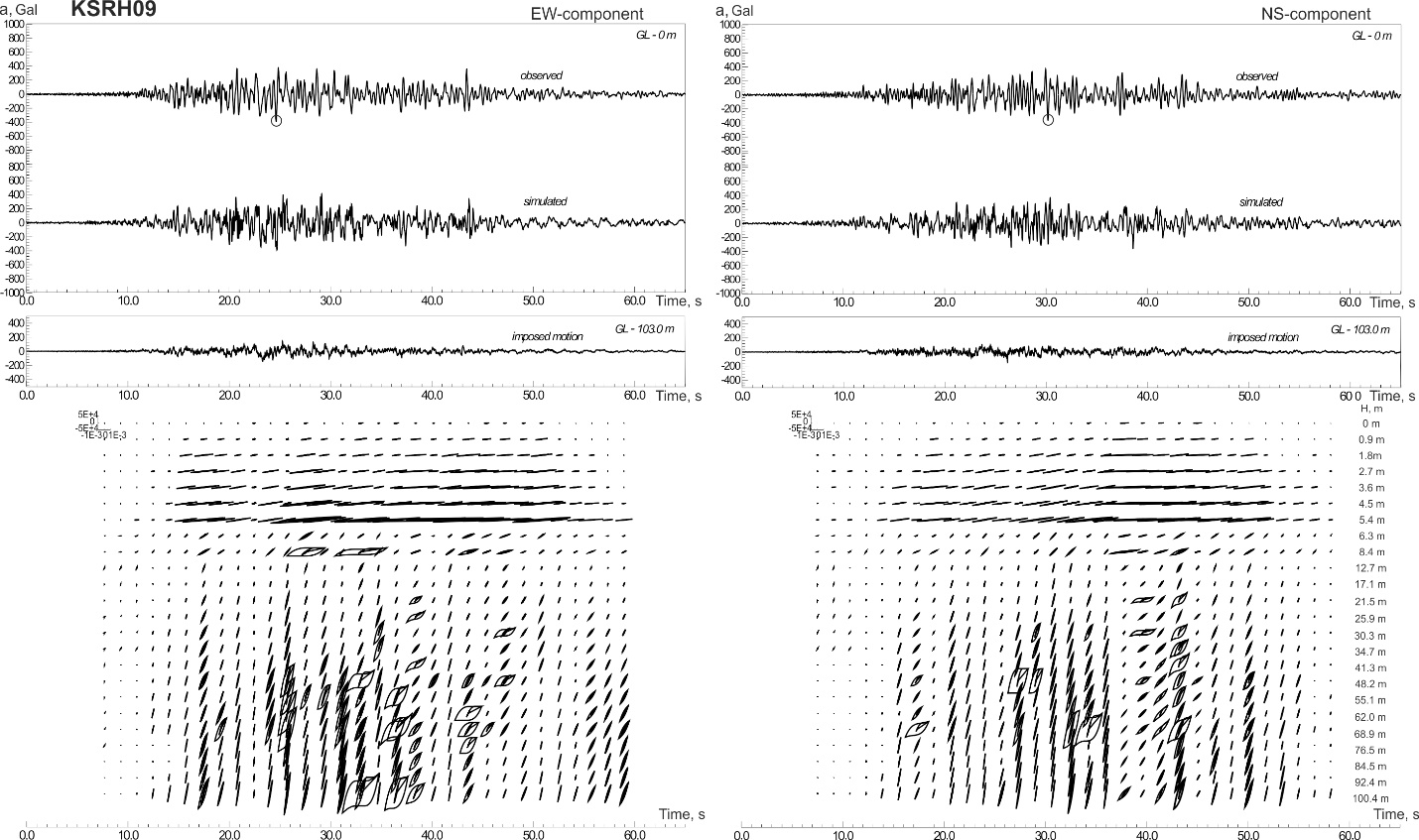


a


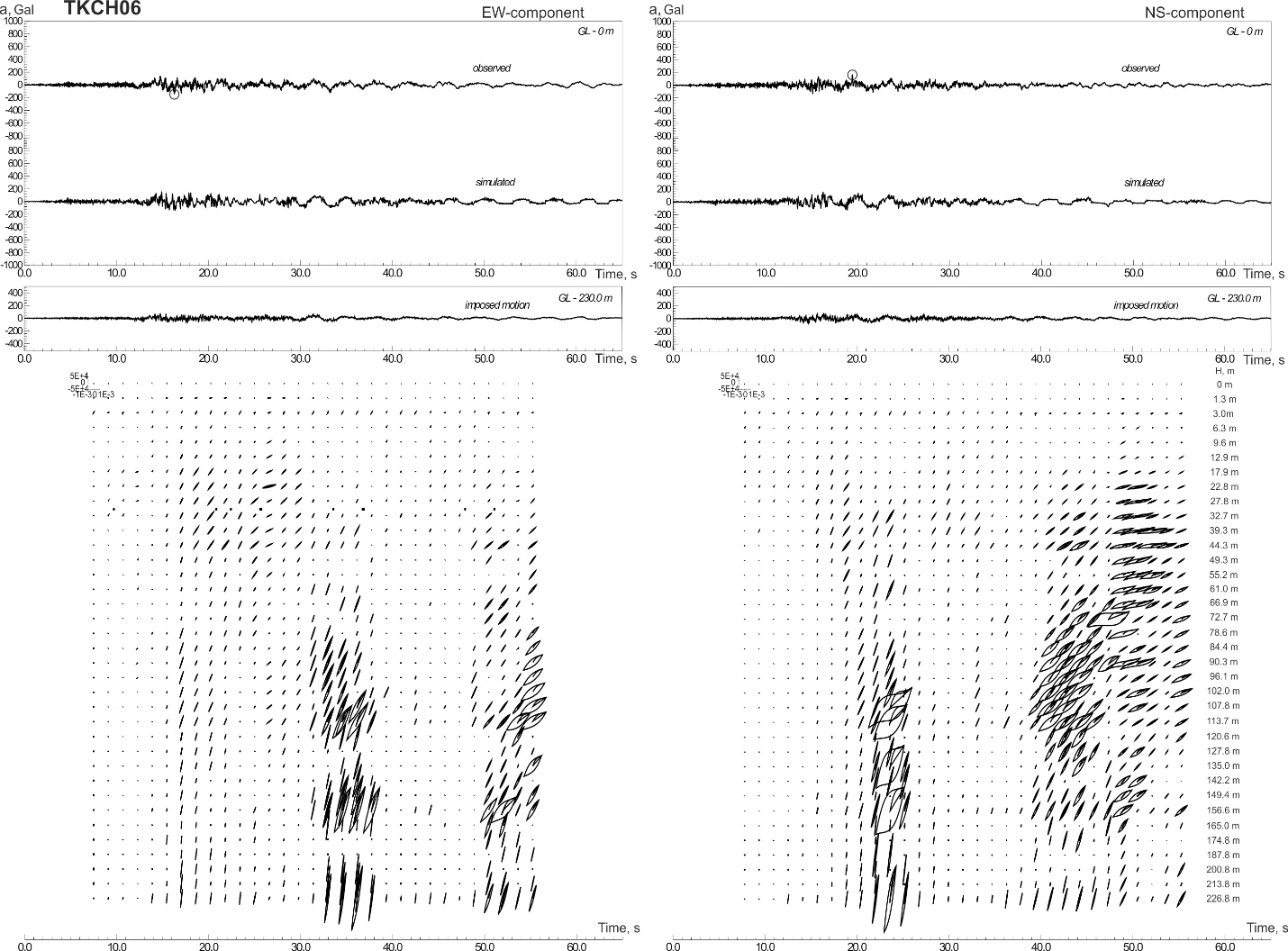


b


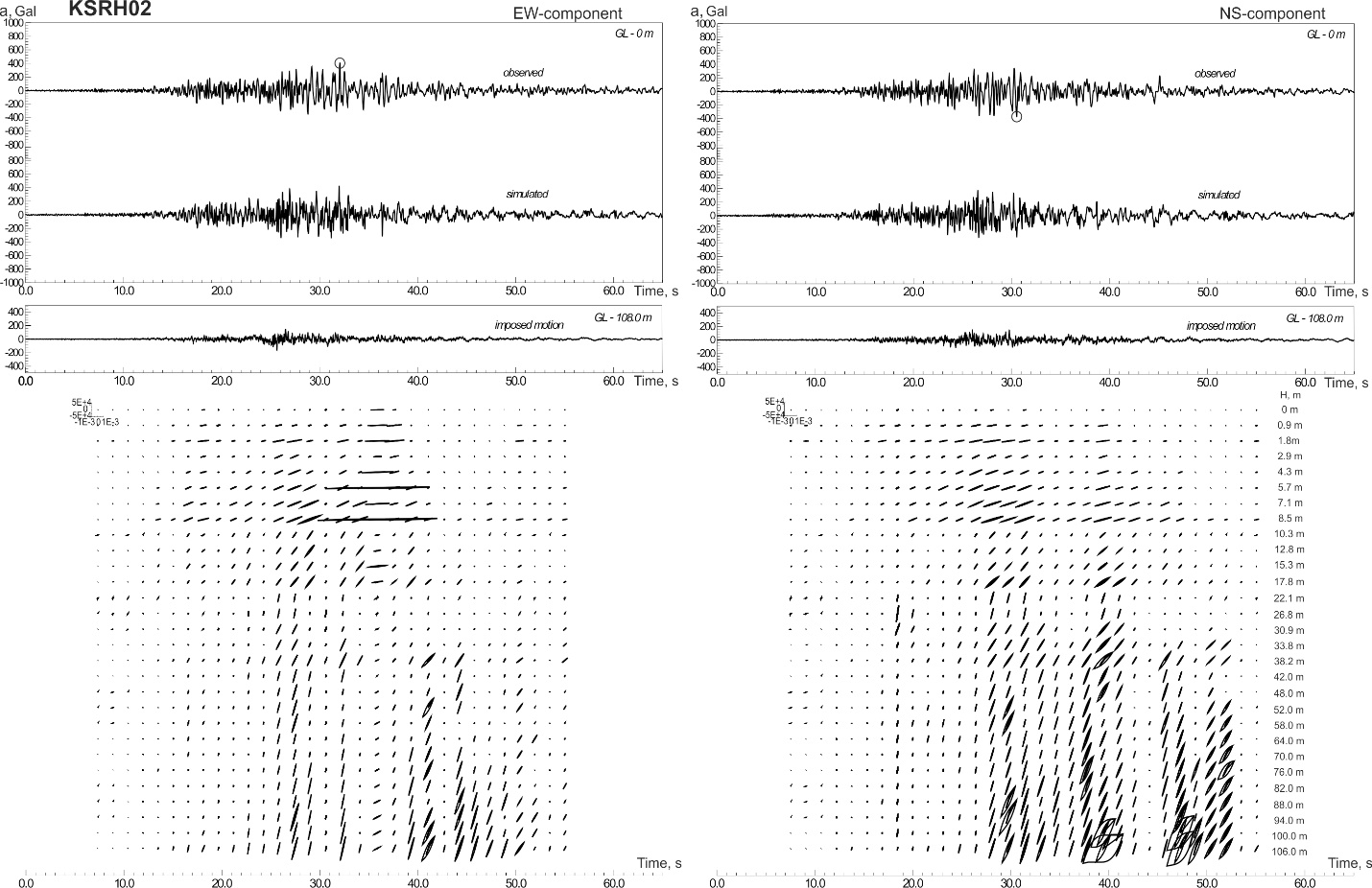


c


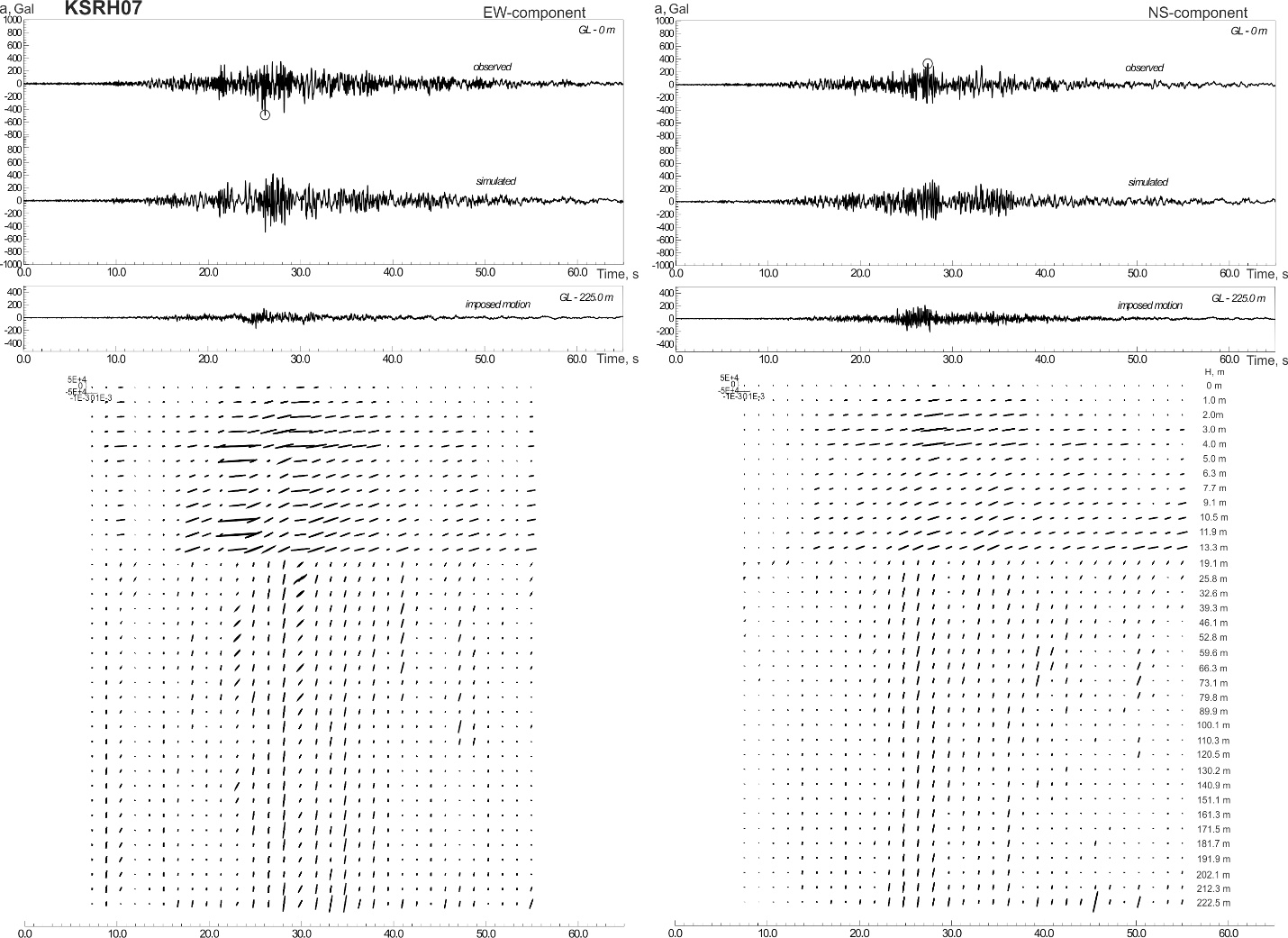


d


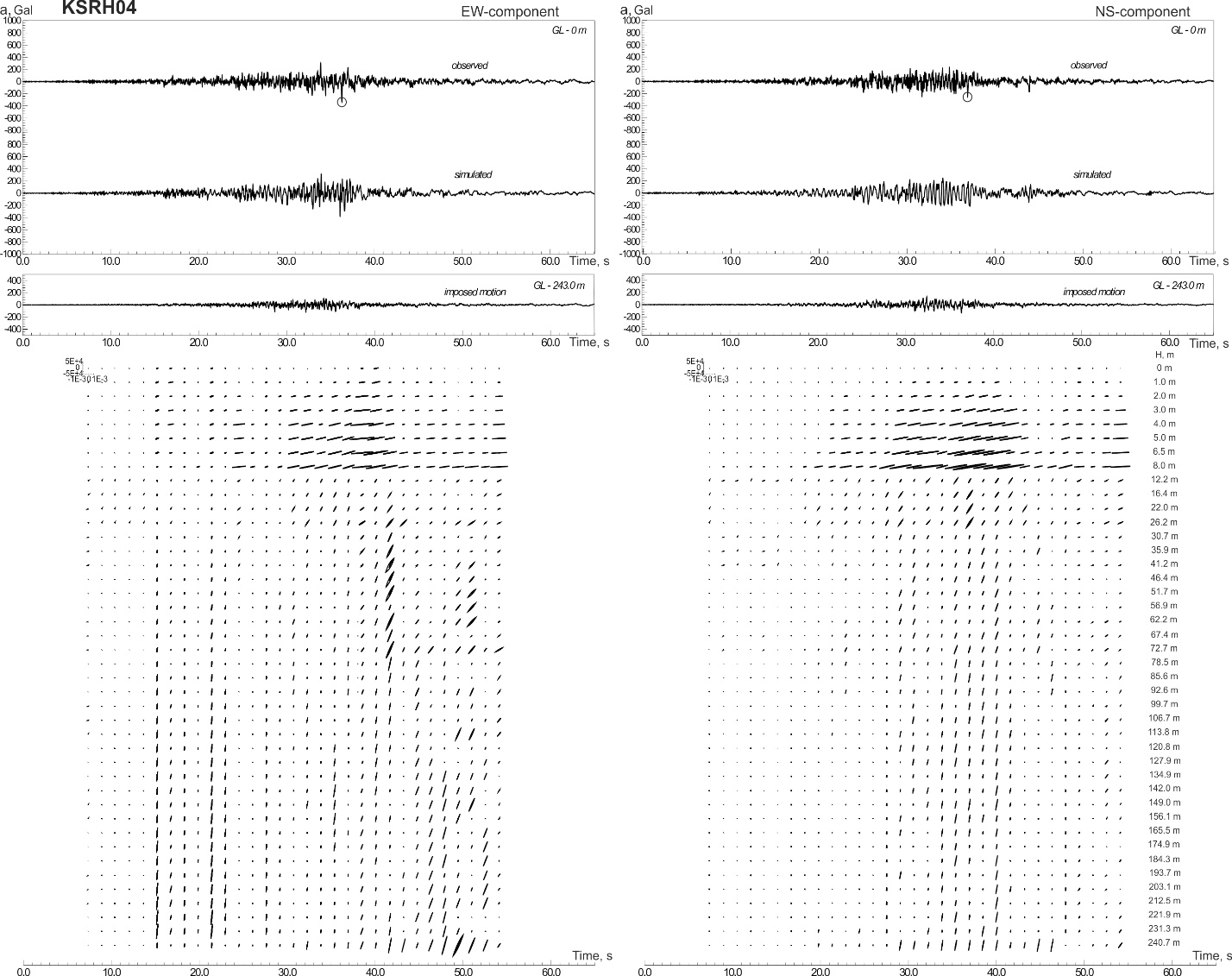


e


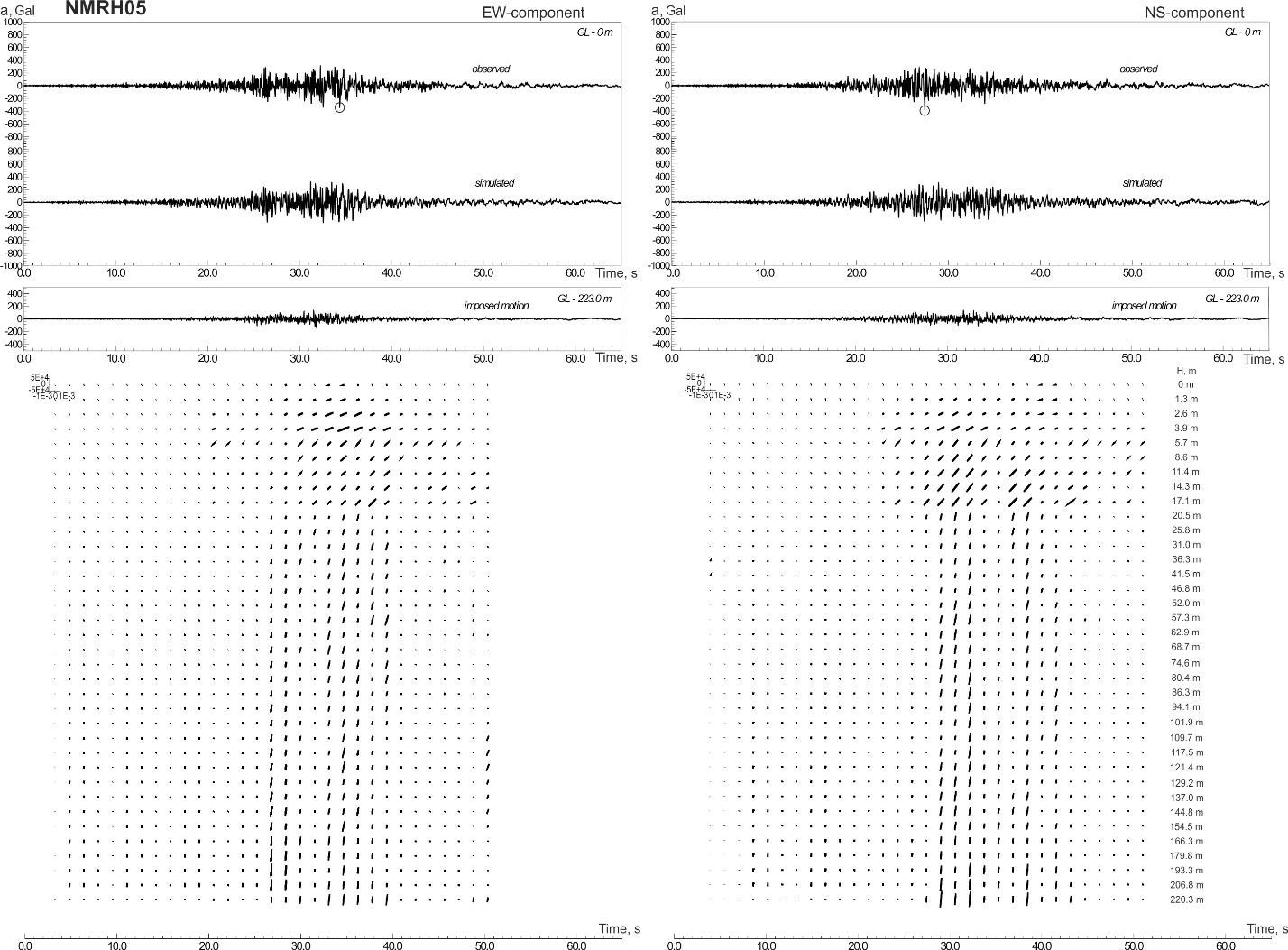


f


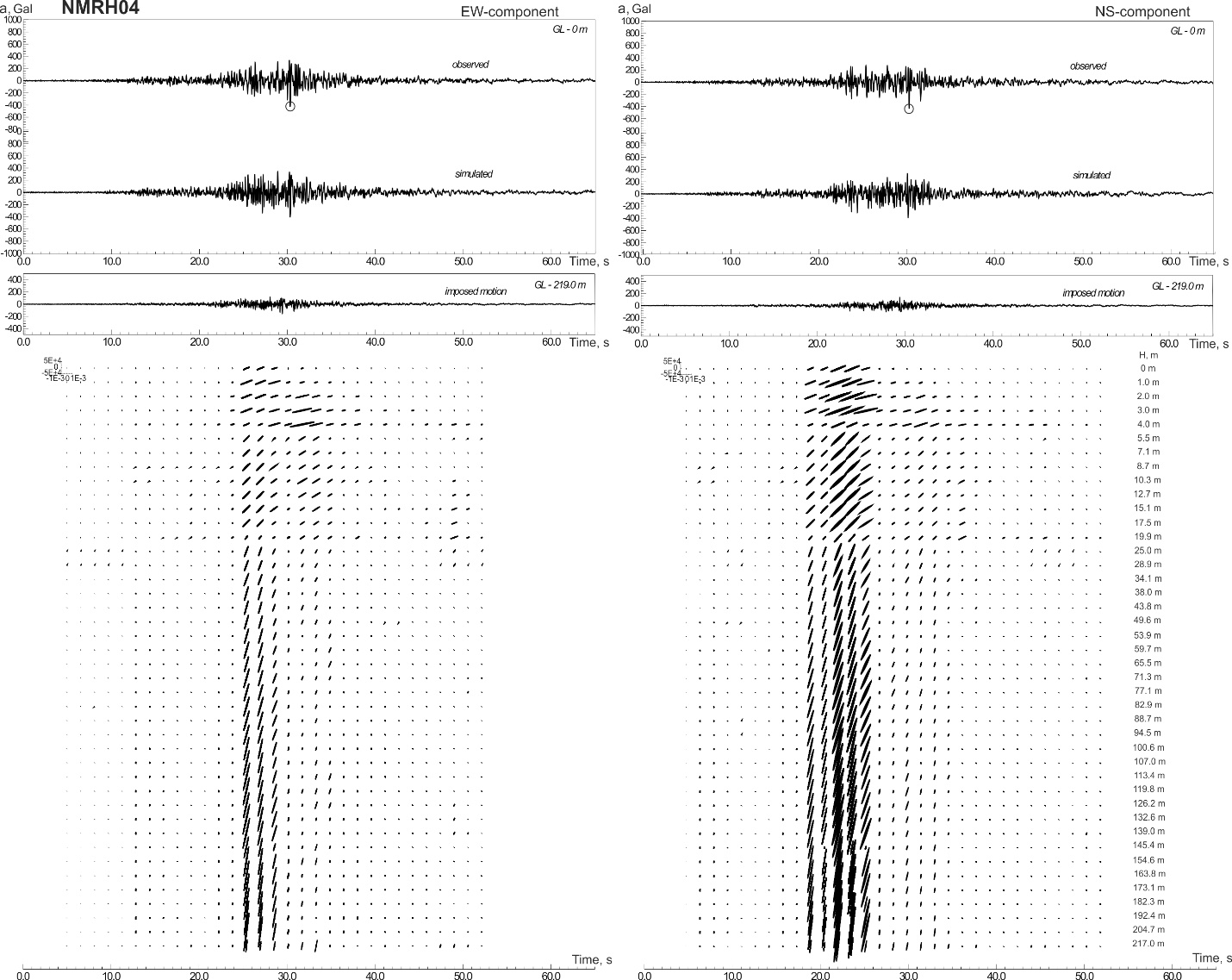


g


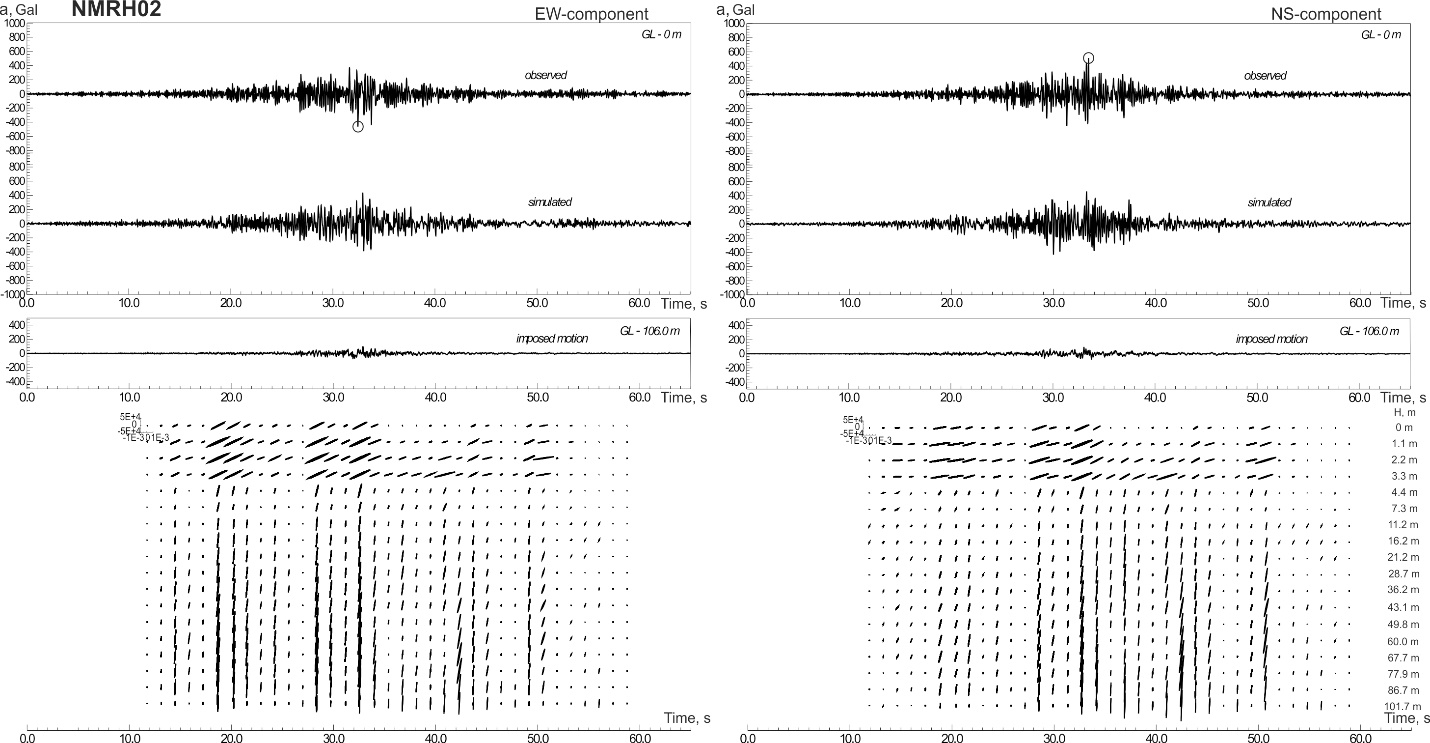


h

Figure S1. Acceleration time histories of the 2003 Tokachi-oki earthquake, observed and simulated, and estimated stress-strain relations in soil layers, changing with time during strong motion: a - at KSRH09 site; b - at TKCH06 site; c - at KSRH02 site; d - at KSRH07 site; e - at KSRH04 site; f - at NMRH05 site; g - at NMRH04 site; h - at NMRH02 site. Stresses are given in Pa, strains in strain. Peak ground accelerations are marked by small circles.


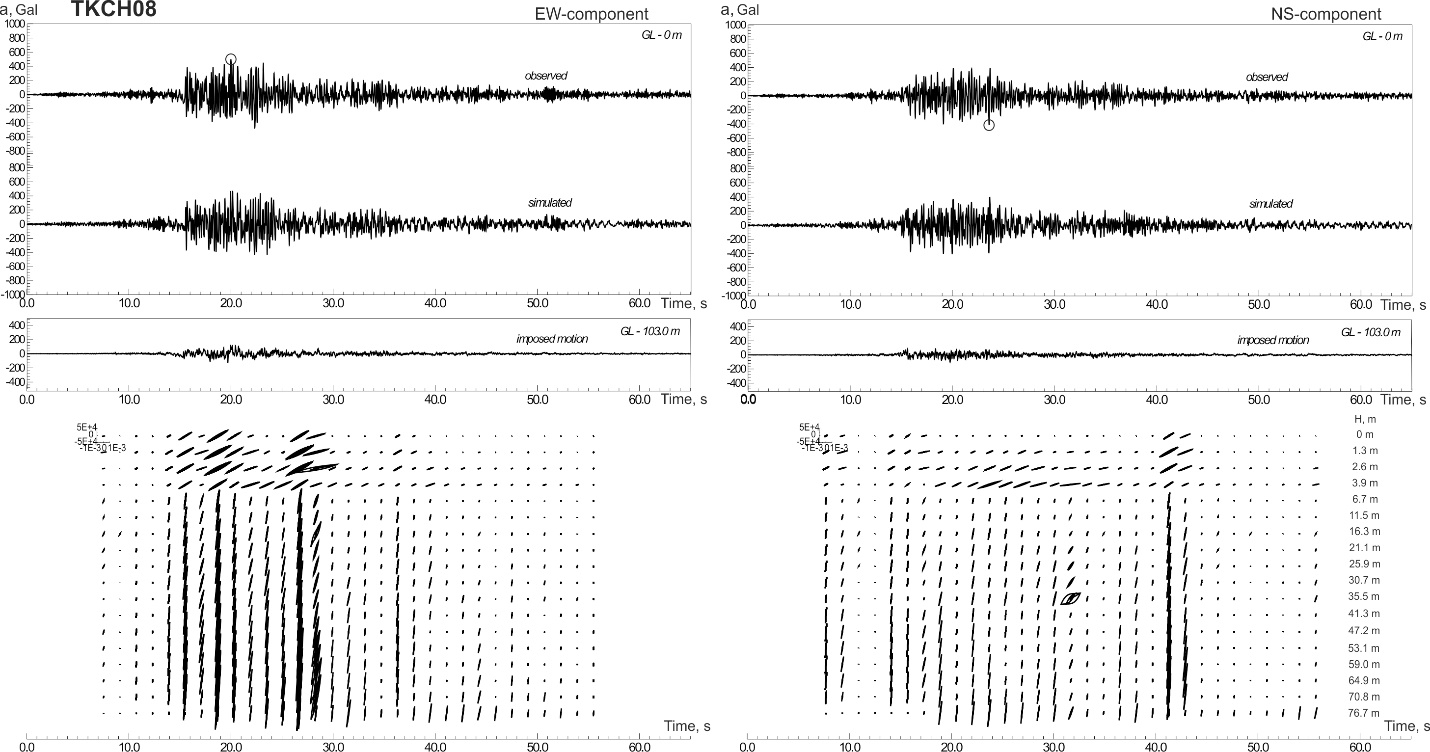
a


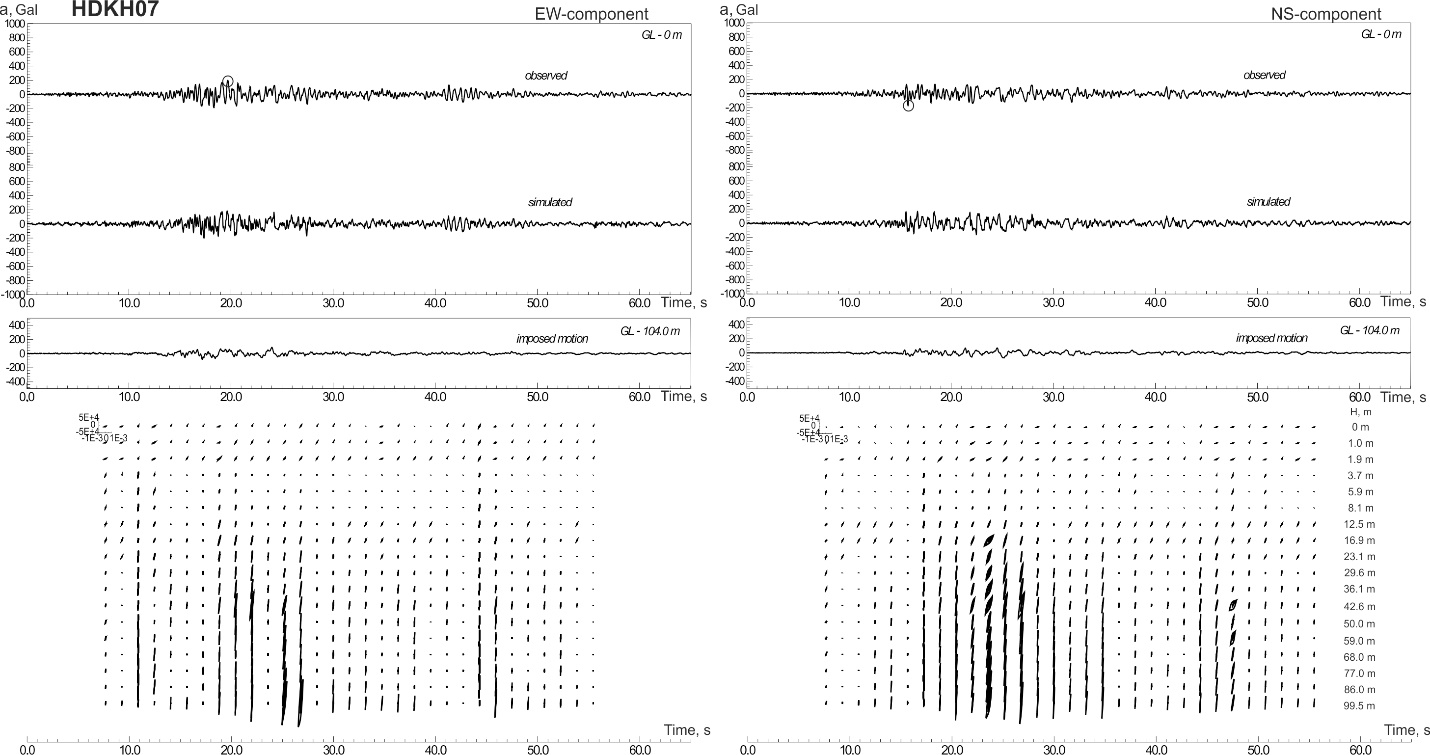


b


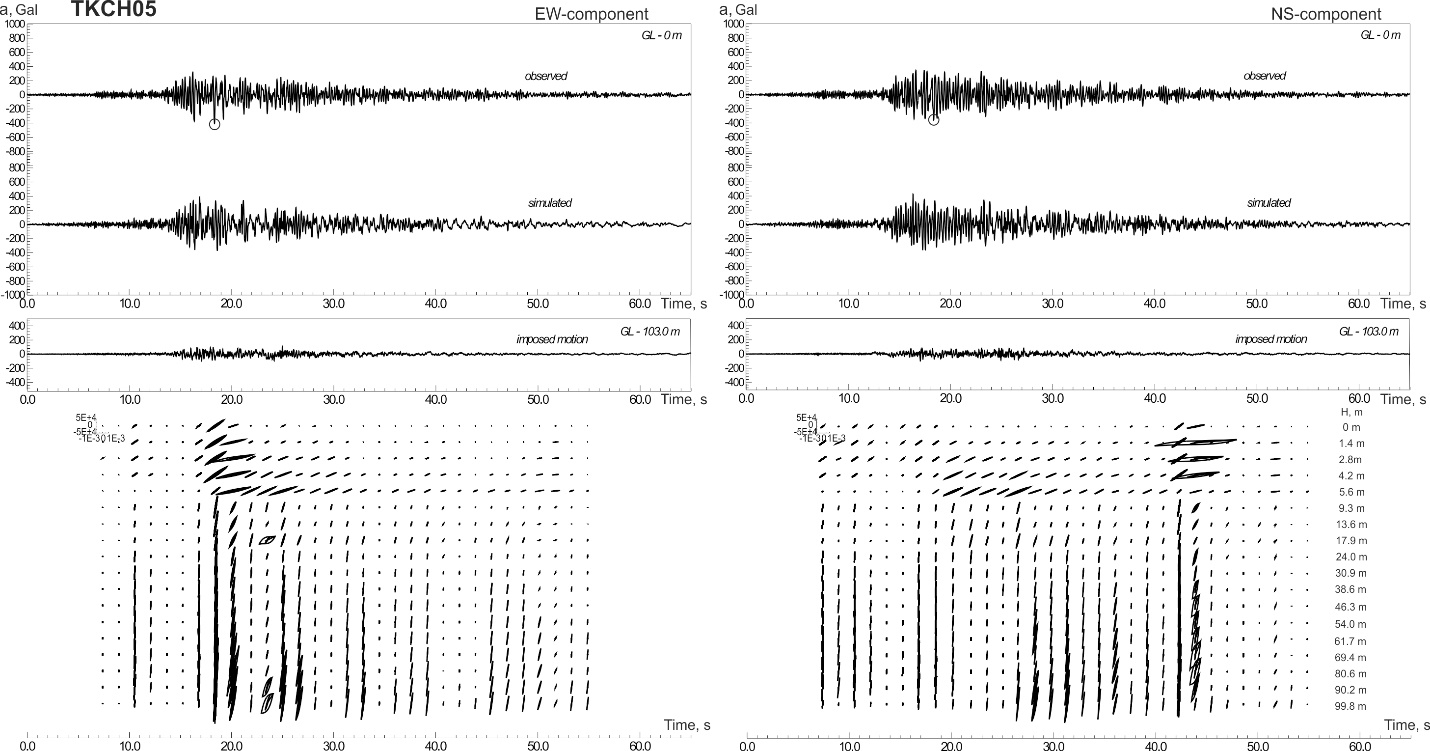


c


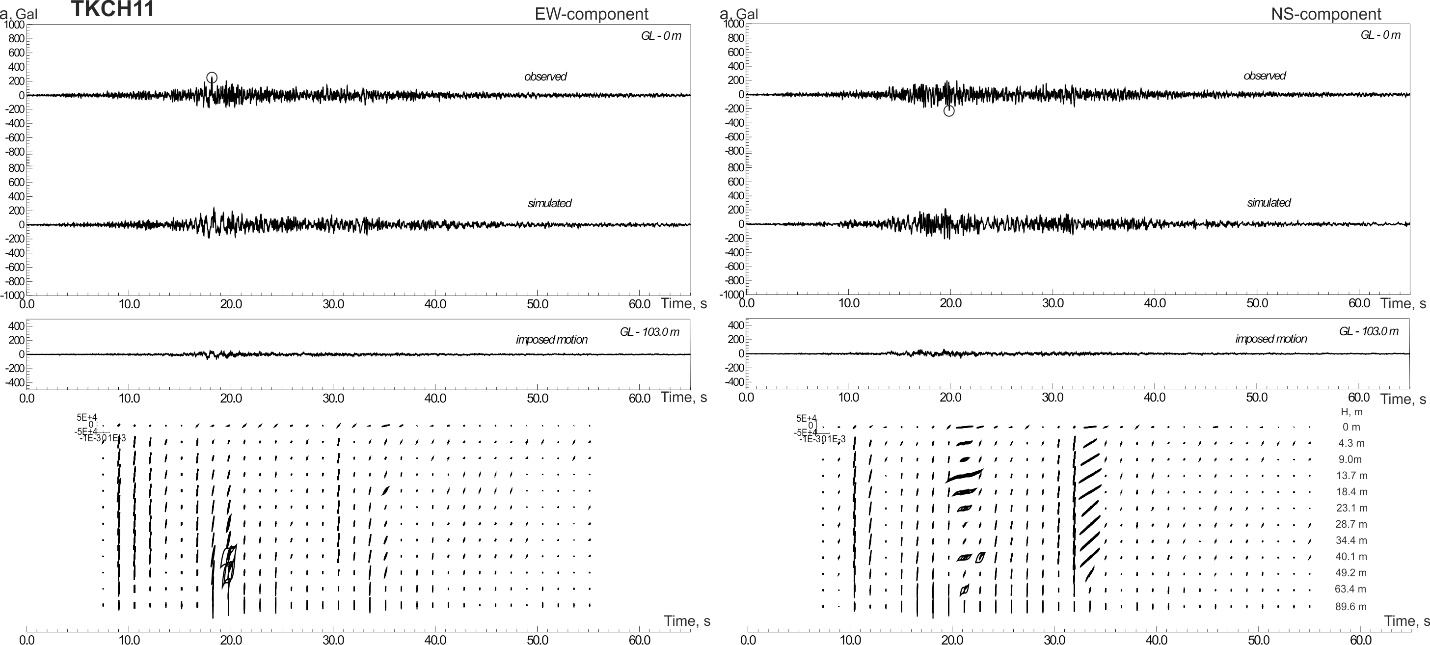


d


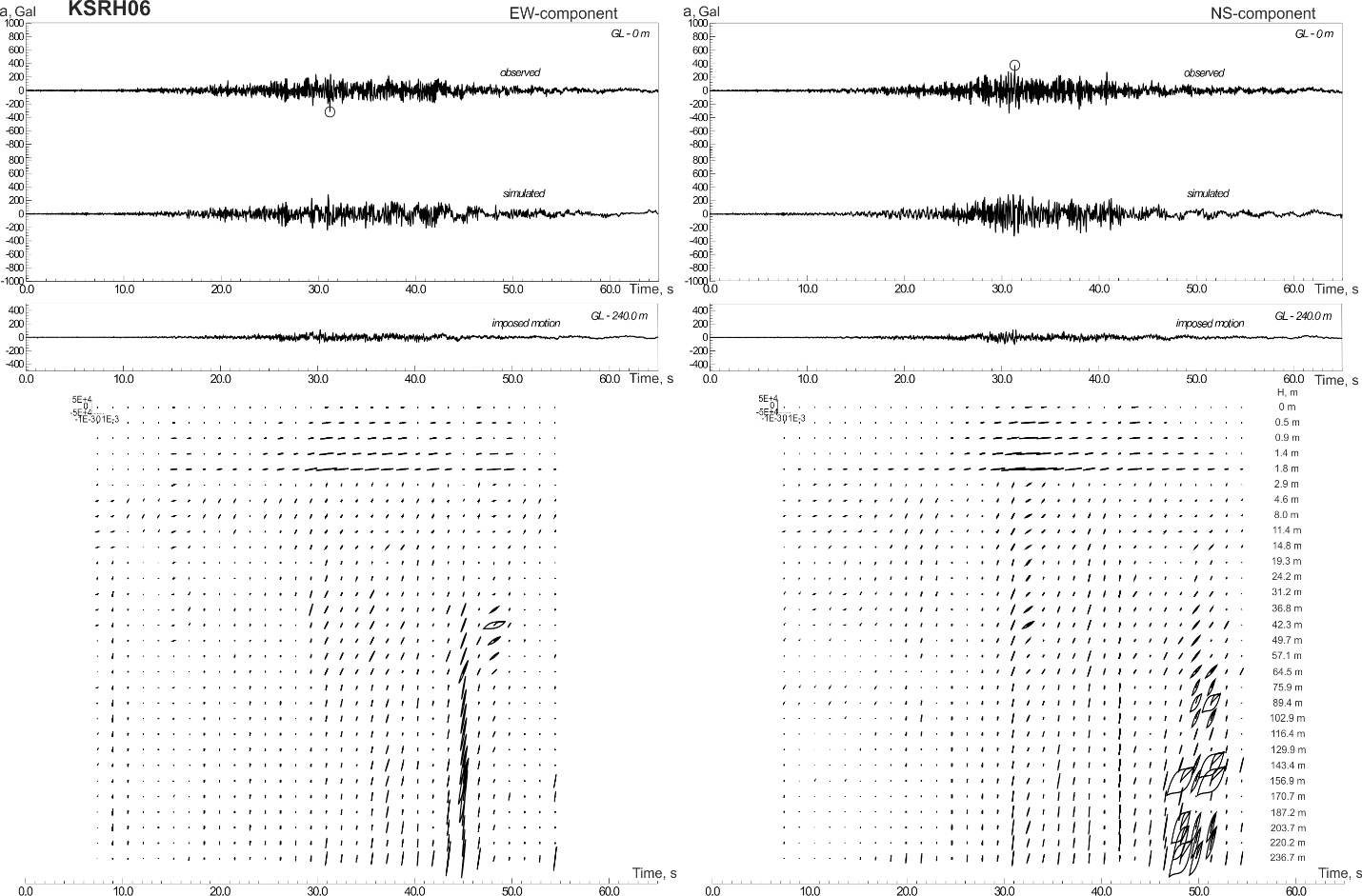


e


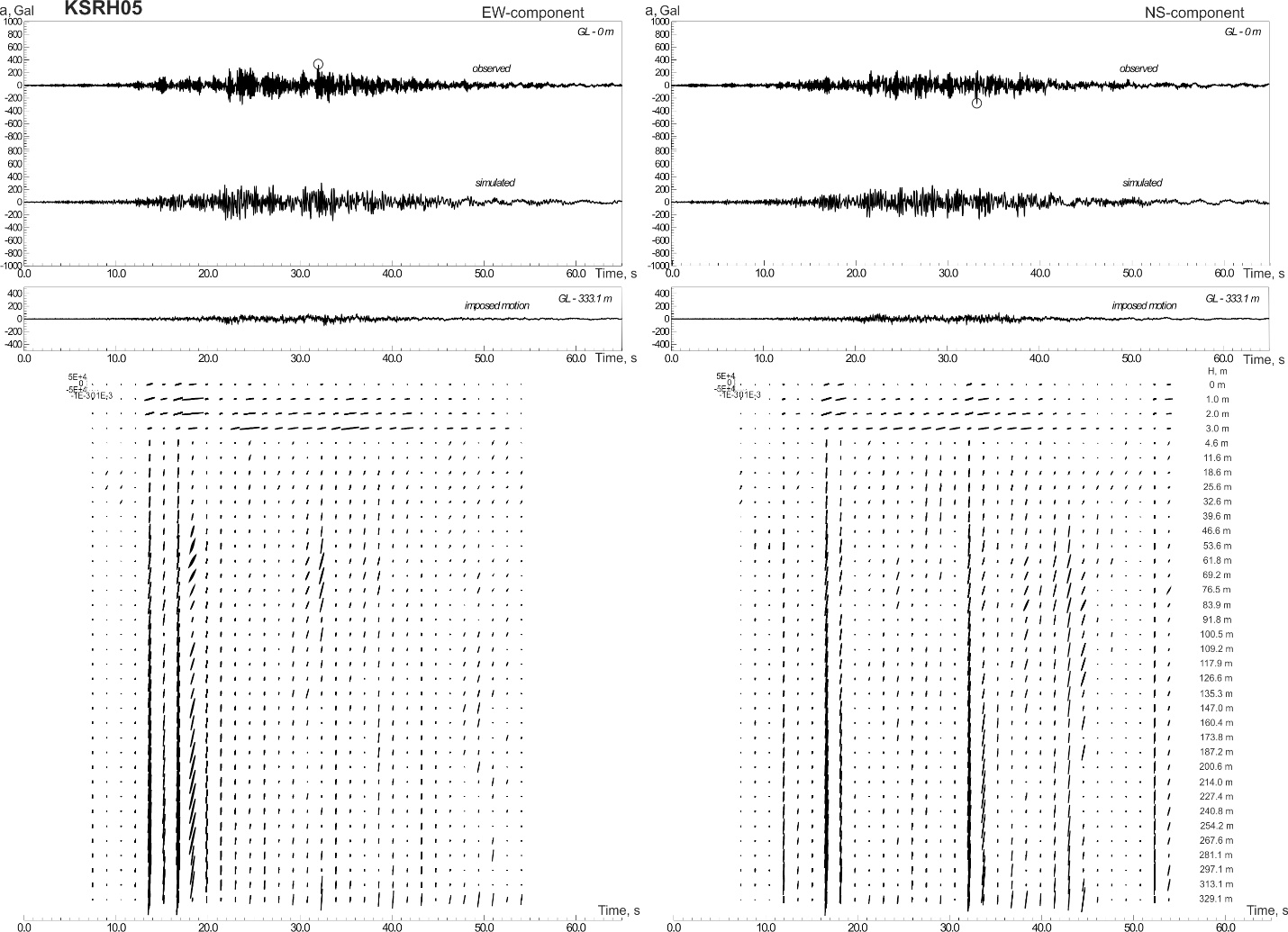


f

Figure S2. Acceleration time histories of the 2003 Tokachi-oki earthquake, observed and simulated, and estimated stress-strain relations in soil layers, changing with time during strong motion: a - at TKCH08 site; b - at HDKH07 site; c - at TKCH05 site; d - at TKCH11 site; e - at KSRH06 site; f - at KSRH05 site. Stresses are given in Pa, strains in strain. Peak ground accelerations are marked by small circles.


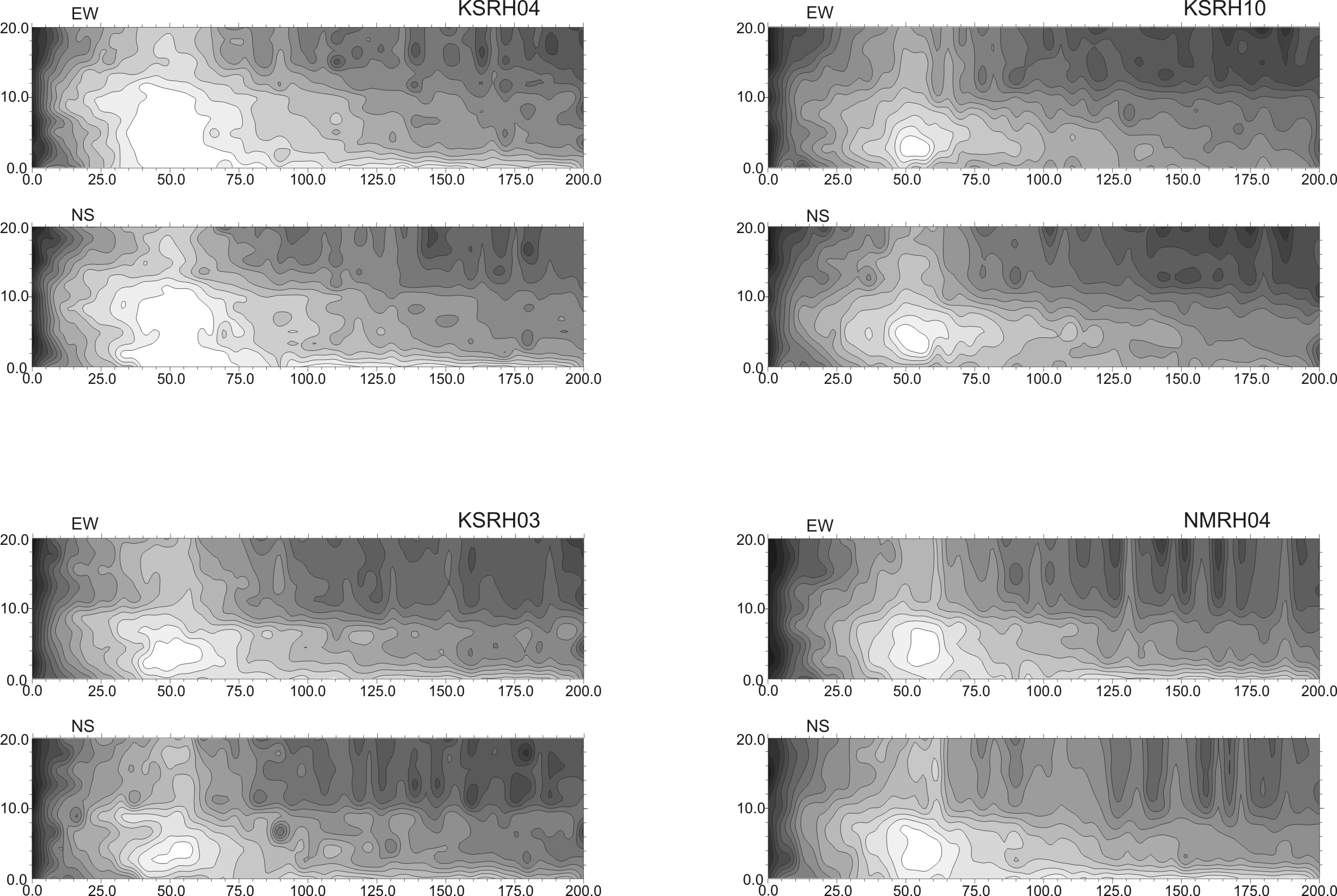


Figure S3. Time-frequency diagrams of the acceleration time histories of the 2003 Tokachi-oki earthquake at KSRH04, KSRH03, KSRH10, and NMRH04 sites at two horizontal components.
